# Demographic Confounding in Voice-Based Parkinson Disease Screening: Methodological Analysis of the Bridge2AI Voice Dataset

**DOI:** 10.2196/95609

**Published:** 2026-08-20

**Authors:** Shikhar Shukla, Parvati Naliyatthaliyazchayil, Judy W Gichoya, Saptarshi Purkayastha

**Affiliations:** 1 Department of Biomedical Informatics and Engineering, Luddy School of Informatics, Computing, and Engineering, Indiana University, 535 W Michigan St., IT 475J, Indianapolis, IN, 46202, United States, 1 3172740439; 2 Department of Radiology and Imaging Sciences, Emory University School of Medicine, Emory University, Atlanta, GA, United States

**Keywords:** confounding factors, voice biomarkers, Parkinson disease, age factors, audio spectrogram transformer, Bridge2AI, speech acoustics, digital health, validation studies, dementia

## Abstract

**Background:**

Voice-based deep learning models for Parkinson disease (PD) and dementia screening report areas under the curve (AUCs) of 0.85‐0.97, but rarely audit demographic confounding. Because speech changes substantially with age, case-control age imbalance alone can produce high classification performance independent of disease.

**Objective:**

We audited the Bridge to Artificial Intelligence (Bridge2AI) Voice Dataset v3.0.0 with three objectives: (1) quantify age and site confounding in voice screening for PD and dementia, (2) evaluate whether a disease-specific acoustic signal persists after demographic adjustment, and (3) propose minimum reporting standards.

**Methods:**

We fine-tuned an audio spectrogram transformer (AST; 86.4 million parameters) using 5-fold participant-level cross-validation for PD (n=253) and dementia (n=221). Logistic regression on age and sex provided a demographic-only baseline under identical splits. Confounding was assessed by (1) restricting evaluation to ages 60‐80 years, (2) restricting to US participants because all Canadian PD (n=62) and all Canadian dementia (n=70) participants were cases with no Canadian controls, (3) retraining AST from scratch on the age-restricted subgroup, (4) 1:1 nearest-neighbor propensity-score matching on age and sex in the US age-restricted PD subgroup, and (5) applying v3.0.0-trained models to v2.0.1 spectrograms of the same participants.

**Results:**

On the full cohort, age alone was statistically indistinguishable from the AST (PD: AUC 0.875 vs 0.843, DeLong *P*=.36; dementia: 0.905 vs 0.895, *P*=.75), indicating a demographic shortcut; because both cohorts share the same 148-person control group, this parallel pattern is one dataset-level artifact, not 2 independent confirmations. Within ages 60‐80 years, the AST exceeded age-only for both conditions (PD: 0.787 vs 0.568, ΔAUC +0.225, 95% CI +0.089 to +0.361; *P*=.001, n=129; dementia: 0.809 vs 0.598, ΔAUC +0.215, 95% CI +0.056 to +0.375; *P*=.008, n=95), though the dementia result reflects residual site confounding. Three convergent estimates of PD-specific signal, post hoc age-restricted (0.787), de novo retrained (0.798), and propensity-matched (US, 28 pairs; 0.760; *P*=.02 vs age-only), all fell substantially below the 0.843 full-cohort AUC. Under the most conservative adjustment (US-only, ages 60‐80 years), AST AUC was 0.726, which we consider the least confounded estimate. Cross-version preprocessing testing produced AUC 0.618, indicating brittle representations. Of 13 published voice-PD studies, only 7 reported case and control age distributions, and none reported a demographic-only baseline or an age-restricted evaluation.

**Conclusions:**

Demographic confounding dominates full-cohort voice-screening metrics on the Bridge2AI Voice Dataset for both PD and dementia. After age restriction, site control, and propensity matching, residual AST performance for PD (AUC 0.726‐0.798) remained significantly above demographic baselines, supporting a disease-specific acoustic signal substantially weaker than full-cohort metrics suggest; for dementia, insufficient US cases precluded similar estimates. Voice biomarker studies should report case and control demographic distributions, demographic-only baselines under identical splits, and age-restricted performance alongside full-cohort metrics.

## Introduction

Voice-based screening for neurological disorders has emerged as a promising noninvasive approach, with deep learning models reporting increasingly high classification accuracy for conditions including Parkinson disease (PD) [1-3]. Speech reflects the coordinated activity of motor, cognitive, and affective neural systems [4], and subtle changes in voice quality, articulation, and prosody have been associated with many health conditions [5,6]. Recent studies applying audio spectrogram transformers (ASTs) [7,8], self-supervised models [9,10], and convolutional architectures [11,12] to PD classification have reported areas under the curve (AUCs) ranging from 0.85 to 0.97, fueling optimism about clinical translation. The AST [7] is a vision transformer adapted for audio classification, pretrained on AudioSet (2 million clips and 527 classes). It has achieved state-of-the-art results on audio classification benchmarks and has been applied to pathological voice detection [8,11].

However, these performance metrics may be systematically inflated by a confounder that is rarely examined: age. PD is predominantly a disease of aging, with onset typically occurring at approximately 60 years of age [13]. At the same time, the human voice undergoes substantial age-related changes, including decreased pitch, increased jitter and shimmer, reduced harmonic-to-noise ratio, and altered vocal fold dynamics [14,15], that are independent of any neurological pathology. When PD cohorts are constructed by comparing older patients against younger healthy volunteers, as is common in convenience-sampled datasets, any classifier (including one with no access to speech at all) may achieve high discrimination simply by exploiting the age gap. This problem is well recognized in medical imaging [16] but has received remarkably little attention in the voice biomarker literature.

The absence of demographic confounding analysis in voice-based PD screening represents a critical methodological gap [17]. While Brenner et al [18] demonstrated that age-based selection bias in the mPower dataset inflates PD classification scores, and Ozbolt et al [19] identified age imbalance between groups as a key methodological concern in sustained-vowel analyses, no study has systematically quantified demographic confounding across multiple conditions on a modern multisite dataset or proposed minimum reporting standards. Most published studies report overall classification metrics without controlling for, or even reporting, the age distribution of cases and controls. Studies that do report demographics often show substantial age imbalances [8,20] but do not assess their impact on performance; for example, Rahman et al [21] reported an 8-year age gap between PD and controls and performed age-trimming (excluding participants younger than 50 years) but did not test a demographic-only baseline or perform age-restricted evaluation (Table S12 in Multimedia Appendix 1).

The severity of this gap is underscored by how readily the confounding can be detected. This study originated from the CIMDAR-HIVE (Developing a Hive Learning and Datathon Supported Course on Imaging and Multimodal Data for Resource-Limited Institutions) training program under the National Institutes of Health (NIH) Common Fund Data Ecosystem initiative, where the first and senior authors designed bias investigation exercises for the 2025 Emory Health AI Summer School & Datathon [22]. During these sessions, participants with no prior experience in voice biomarker research identified age confounding in the Bridge to Artificial Intelligence (Bridge2AI) dataset within hours, a finding that, despite its simplicity, has not been reported or addressed in any published study using this corpus. The formal analysis presented here systematically investigates that initial observation.

This study addresses this gap directly using the Bridge2AI Voice Dataset v3.0.0 [23,24], a publicly available corpus of 833 participants across 5 clinical sites. We fine-tuned an AST [7] for PD and dementia screening under strict participant-level cross-validation, and then systematically investigated the role of demographic confounding through age-only baselines, age-restricted subgroup evaluation, site-level analysis, and cross-version preprocessing robustness testing. While demonstrated on a single dataset, the demographic structure we identify is common to many voice datasets; we argue that age-restricted subgroup evaluation should become a standard reporting requirement for voice-based disease screening research.

## Methods

### Overview

**Figure 1.**
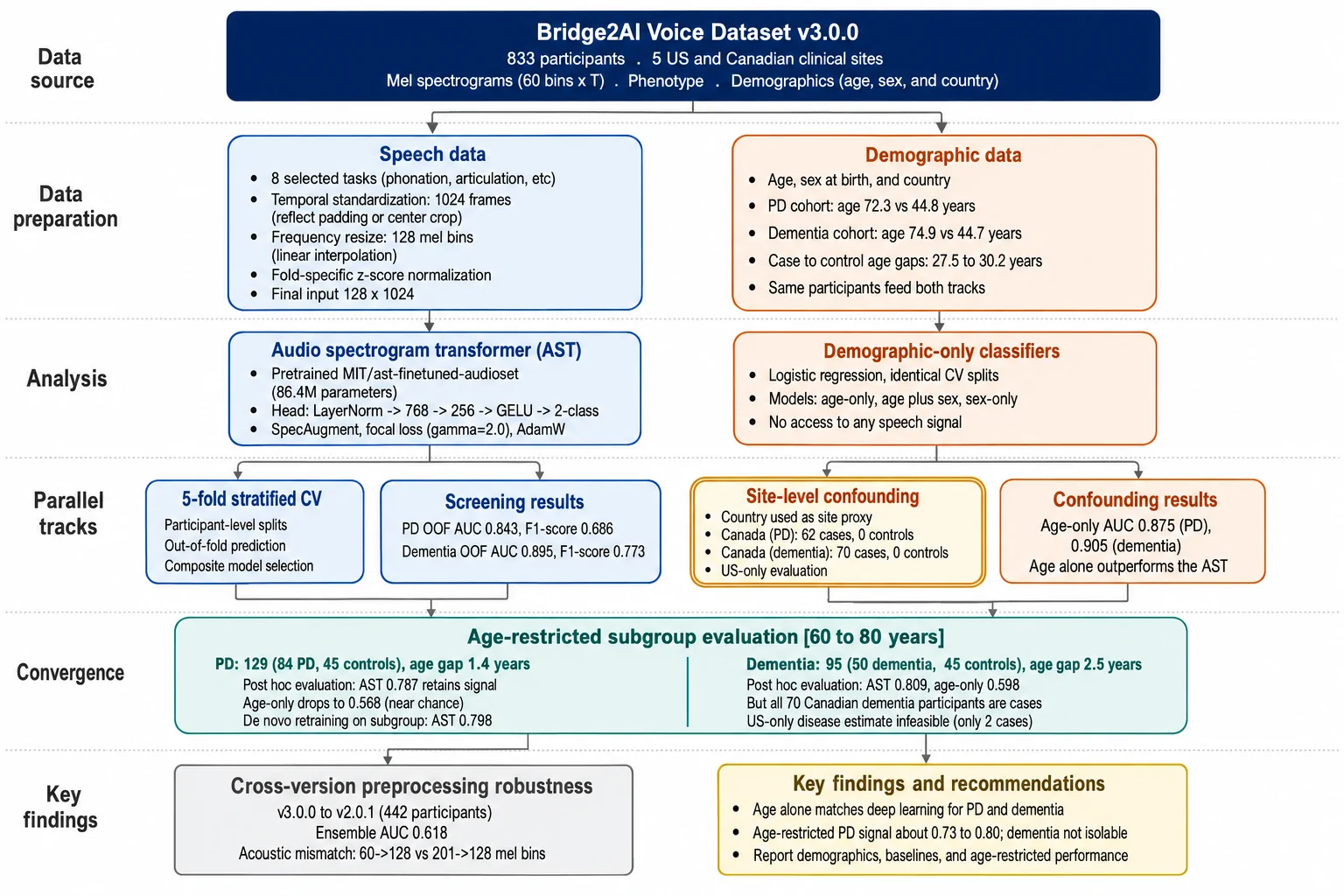
Methodology overview of the demographic confounding analysis framework. AST: audio spectrogram transformer; AUC: area under the curve; Bridge2AI: Bridge to Artificial Intelligence; CV: cross-validation; GELU: Gaussian Error Linear Unit; OOF: out-of-fold; PD: Parkinson disease.

We developed a transformer-based speech screening framework (Figure 1) using spectrogram representations and a pretrained AST, applied to the publicly available Bridge2AI Voice Dataset v3.0.0. The same architecture, preprocessing, training procedure, and evaluation protocol were applied to both PD and dementia; only the resulting cohort compositions differed (Table 1). Beyond the confounding analysis, we evaluate the AST for dementia screening, compare spectrogram-based and phonetic posteriorgram (PPG) [25] representations, assess baseline models, and examine the effect of speech task selection on screening performance. Depression screening (44 cases) was conducted as an exploratory analysis reported in Multimedia Appendix 1.

**Table 1.** Selected speech tasks for PD^a^ and dementia screening (Bridge2AI^b^ voice dataset v3.0.0; 833 participants; 5 clinical sites)^c^.

Condition	Tasks	Participants (cases and controls)	Recordings	Case (%)
Parkinson disease	8^d^	253 (106, 147)	2131	41.9
Dementia	8^d^	221 (73, 148)	1791	33

a PD: Parkinson disease.

b Bridge2AI: Bridge to Artificial Intelligence.

c Prolonged vowel; glides (high-to-low, low-to-high); diadochokinesis (pataka); rainbow passage; picture description; story recall; maximum phonation time.

d Eight tasks spanning phonation, articulation, and cognitive-linguistic domains were selected based on case and control representation.

To assess demographic confounding, we additionally trained a logistic regression classifier using only age and sex as features, with identical cross-validation splits. Age-restricted subgroup analyses were performed both post hoc (evaluating existing out-of-fold [OOF] predictions on age-restricted subsets) and prospectively (retraining the AST from scratch on the age-restricted subgroup). Country-level analysis was conducted as a proxy for multisite evaluation, and attention map analysis was performed to characterize the spectrotemporal features driving classification. Full details of all supporting analyses are provided below.

### Dataset Description

This study used the Bridge2AI Voice Dataset v3.0.0 [23,24], a publicly available corpus comprising 833 participants with voice recordings from individuals with neurological and psychiatric conditions as well as healthy controls, collected across 5 clinical sites. The dataset includes multiple structured speech tasks designed to elicit a broad range of vocal and speech characteristics, such as sustained phonation, pitch control, articulatory agility, connected speech, and cognitive-linguistic tasks [24].

Voice recordings were provided in the form of log-Mel spectrograms, stored in Parquet format (*features/torchaudio_mel_spectrogram.parquet*). Each spectrogram consisted of 60 Mel frequency bins and a variable number of time frames, reflecting differences in recording duration across tasks and participants. Additionally, v3.0.0 provides PPGs extracted from a pretrained neural speech model [25], stored in the ppgs.parquet file in the features folder, with 40 phoneme classes and variable temporal length.

Participant-level phenotype data were obtained from the hierarchical phenotype annotations under the diagnosis subdirectory of the phenotype directory. PD cases (106 participants) were identified from *parkinsons_disease.tsv*, dementia cases (73 participants) from *cognitive_impairment.tsv*, and controls (148 participants) from *control.tsv*. One participant appearing in both PD and control files was assigned to the PD group. Spectrograms and PPGs were merged with phenotype data using participant identifiers (6-digit numeric, zero-padded).

For cross-version preprocessing robustness testing, Bridge2AI Voice Dataset v2.0.1 was used. v2.0.1 comprises 442 participants with spectrograms of 201 Mel frequency bins, a single phenotype file, and 8-character hexadecimal participant identifiers. Although the identifier formats differ between versions, session-level identifier matching confirms that 441 of 442 v2.0.1 participants reappear in v3.0.0 under reanonymized numeric identifiers, consistent with the PhysioNet release notes describing v3.0.0 as adding “an additional 391 participants” to the prior release. This cumulative-release convention is standard practice for biomedical reference cohorts (eg, UK Biobank, ADNI, and MIMIC), where successive versions extend rather than replace prior data; researchers comparing across Bridge2AI Voice releases should not interpret v2.0.1 and v3.0.0 as independent cohorts. The two versions therefore represent the same participant cohort with different spectrogram extraction pipelines, enabling a controlled assessment of preprocessing sensitivity.

### Task Selection and Inclusion Criteria

To support condition-specific screening while maintaining a unified modeling pipeline, speech tasks were selected based on adequate representation of both cases and controls within the v3.0.0 dataset. Tasks were prioritized according to the number of unique case and control participants with available recordings, ensuring sufficient representation for stable participant-level model training and evaluation.

Eight tasks with adequate PD and control representation were selected: prolonged vowel, glides (high-to-low and low-to-high), diadochokinesis (pataka), rainbow passage, picture description, story recall, and maximum phonation time. These tasks span sustained phonation, pitch control, articulatory agility, connected speech, and cognitive-linguistic domains. The same 8-task set was used for both PD and dementia analyses. Per-task recording distributions are provided in Table S6 in Multimedia Appendix 1. Depression screening used a separate task set selected for case coverage (Multimedia Appendix 1).

Only spectrograms with at least 100 timeframes were retained to ensure sufficient spectrotemporal context for modeling. Table 1 summarizes the selected task sets for each condition.

### Preprocessing

#### Overview

**Figure 2.**
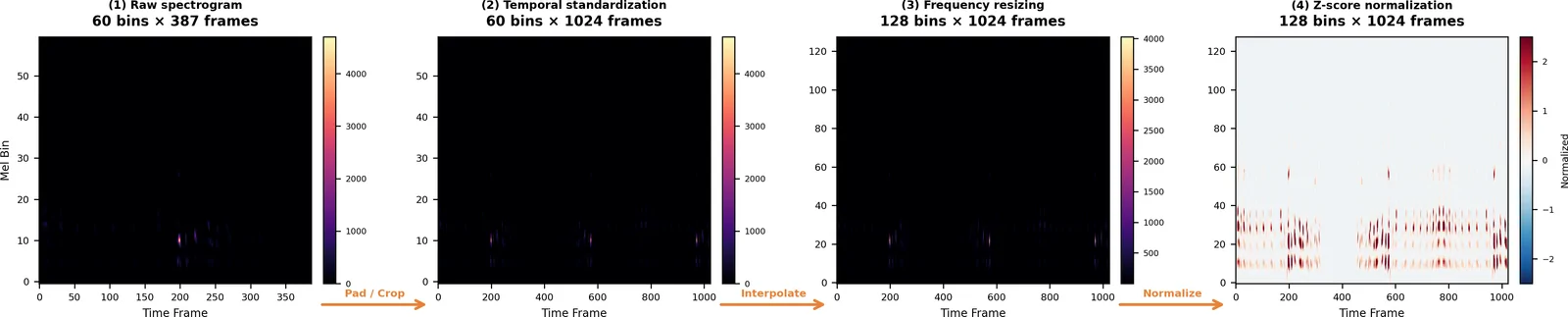
Spectrogram preprocessing pipeline for audio spectrogram transformer input. Example Mel spectrogram from a Parkinson disease participant illustrating sequential preprocessing steps: (1) raw spectrogram with variable temporal length (F × T, where *F*=60 in v3.0.0), (2) center crop or reflective padding to a fixed length of 1024 time frames (F × 1024), (3) interpolation-based resizing to 128 Mel bins (128 ×1024) to match audio spectrogram transformer input requirements, and (4) *z*-score normalization (mean approximately 0, SD approximately 1).

Raw spectrograms (60 Mel bins×variable time frames in v3.0.0; 201 Mel bins in v2.0.1 used for preprocessing robustness testing) were preprocessed to produce fixed-size inputs compatible with the AST architecture. Spectrograms with fewer than 100 timeframes were excluded. This filter operates at the recording level rather than the participant level, so it removes short recordings rather than entire participants; its effect is therefore a subset of the broader task-missingness mechanism discussed below. Temporal length was standardized to 1024 frames using reflect padding (for shorter recordings) or center cropping (for longer ones). The frequency axis was resized to 128 Mel bins via linear interpolation, yielding a uniform 128×1024 representation. Fold-specific z-score normalization was applied, with statistics computed exclusively from each fold’s training partition to prevent information leakage. Representative preprocessed spectrograms are shown in Figure 2. Full preprocessing details, including the rationale for reflect padding over zero padding and the interpolation method, are provided in Multimedia Appendix 1.

#### PPG Preprocessing

PPGs (40 phoneme classes×variable time) were preprocessed identically to spectrograms (reflect-padded or center-cropped to 1024 frames, resized to 128×1024, fold-normalized). We note that interpolating 40-dimensional PPG vectors to 128 dimensions via linear interpolation introduces artifacts, as PPGs represent posterior probabilities over discrete phoneme classes. The interpolated values lack meaningful phonetic interpretation. This preprocessing decision limits the validity of comparisons between PPG-based and spectrogram-based AST performance. A supplementary 1-dimensional (1D) convolutional neural network (CNN) evaluated on native 40-dimensional PPGs is described in Multimedia Appendix 1.

### Cross-Version Preprocessing Robustness

To assess the sensitivity of learned representations to preprocessing changes, the 5-fold models trained on v3.0.0 spectrograms were applied as an ensemble (averaged predicted probabilities) to the v2.0.1 spectrograms of the same participants (442 participants). Because v2.0.1 participants are a subset of v3.0.0 (confirmed via session identifier matching; see the “Dataset Description” section), this test isolates the effect of the feature extraction pipeline (60 vs 201 Mel bins) from population-level differences. Because v3.0.0 spectrograms (60 Mel bins) are upsampled to 128 bins via linear interpolation, while v2.0.1 spectrograms (201 Mel bins) are downsampled to 128 bins, this test conflates preprocessing domain shift with any genuine generalization gap. The resulting AUC should be interpreted as a measure of model sensitivity to the feature extraction pipeline, not as a pure external validation. v2.0.1 spectrograms were resized to 128×1024 and normalized using each fold’s v3.0.0 training statistics. The full protocol is described in Multimedia Appendix 1.

### Participant-Level Data Splitting

All spectrograms from a given participant were assigned exclusively to training or validation, with no participant overlap between partitions. A preliminary 80/20 split was used during pipeline development; all reported results are based on the 5-fold cross-validation described below.

### Model Architecture and Training

The classifier was built on the AST [7], a vision transformer adapted for audio, initialized from AudioSet-pretrained weights (MIT/ast-finetuned-audioset-10-10-0.4593 via Hugging Face). Input spectrograms (128×1024) were divided into nonoverlapping patches with learned positional embeddings, and the full transformer backbone (approximately 86.4 million parameters) was fine-tuned end-to-end. A classification head consisting of layer normalization, a 256-unit dense layer with Gaussian Error Linear Unit (GELU) activation, dropout (rate=0.3), and a 2-class output layer was appended to the pooled transformer output.

SpecAugment-style time and frequency masking was applied during training (50% probability each). Models were trained using AdamW with differential learning rates (backbone 5×10⁻⁶; head 5×10⁻⁴), cosine annealing, focal loss with per-fold inverse class-frequency weights, and gradient clipping. Early stopping was based on a composite of participant-level AUC (weight 0.4) and optimized *F*_1_-score (weight 0.6), evaluated on the held-out validation fold after each epoch, with training terminating after 10 epochs without improvement. Because the epoch-selection criterion was computed on the same held-out fold whose predictions were subsequently aggregated as OOF performance, the reported cross-validated metrics carry an optimization bias; we quantify the direction of this bias in the “Limitations” section. The higher *F*_1_-score weight reflects the clinical priority of balanced sensitivity-specificity over discrimination alone. Full hyperparameter values are provided in Table S1 in Multimedia Appendix 1.

### Evaluation

#### Participant-Level Evaluation

For each condition, predictions were aggregated at the participant level by averaging the predicted probabilities across all recordings from the same participant. Participant-level predictions were computed as unweighted means of recording-level probabilities across all tasks completed by that individual. Because not all participants completed all tasks (recording counts range from 247 to 290 across the 8 selected tasks), this averaging implicitly weights toward the tasks present for each participant. If task completion correlates with age or condition, for example, if older or more impaired participants systematically fail to complete cognitively demanding tasks, the aggregated predictions may partially reflect missingness patterns rather than acoustic features. Performance was evaluated using AUC-ROC (area under the receiver operating characteristic curve; threshold-independent) as the primary metric, supplemented by *F*_1_-score, precision, and recall. To prevent test-set leakage in threshold selection for the secondary metrics, optimal thresholds were determined per fold using a leave-one-fold-out (LOFO) Youden J procedure: for each fold k, the threshold was computed from the OOF predictions of the remaining 4 folds and applied to fold-k validation predictions. This ensures that the threshold for any participant’s classification is independent of that participant’s prediction. For comparison, results at a fixed threshold of 0.5 and at training partition–derived thresholds are reported in Multimedia Appendix 1. Pairwise differences in AUC between models evaluated on the same participants were tested using the DeLong test, which yields exact 95% CIs for the AUC difference and accounts for the correlation induced by paired predictions. Because 9 AST-versus-demographic-baseline comparisons were performed across the full and age-restricted cohorts for both conditions, *P* values were adjusted for multiple comparisons using the Benjamini-Hochberg false discovery rate (FDR) procedure; both raw *P* values and adjusted q-values are reported. This ensures that evaluation reflects participant-level classification rather than individual recording-level performance.

#### Five-Fold Cross-Validation

To assess model robustness for each condition, 5-fold stratified cross-validation was performed at the participant level using a fixed random seed (random state=42), ensuring that each participant contributed to validation in exactly 1-fold. Cross-validation was performed over all participants in each condition-specific cohort, not restricted to the training partition described in the “Participant-Level Data Splitting” section. Within each fold, a fresh AST model was initialized from the same pretrained weights and trained with the procedure described above. Fold-specific normalization statistics were computed from the training partition of each fold and applied to the corresponding validation partition. Participant-level AUC-ROC, *F*_1_-score, recall, and precision were computed for each fold, and OOF predictions were aggregated across all 5 folds to report true cross-validated performance. Mean and SD of per-fold metrics are reported. Bootstrap 95% CIs (2000 iterations with replacement) were computed from the OOF predictions for key AUC estimates.

#### Demographic Confounding Analysis

To assess demographic confounding, participant-level age and sex at birth were extracted from the demographics file and used to train a logistic regression classifier with balanced class weights. The metadata logistic regression used the same 5-fold cross-validation splits as the AST, applied independently to both the PD cohort (252/253 participants with complete data) and dementia cohort (220/221 participants). OOF predictions from the metadata-only model were compared to the AST’s OOF predictions to quantify the contribution of demographic covariates.

To disentangle age-related voice changes from disease-specific signals, the AST’s existing OOF predictions were evaluated on age-restricted subgroups (55 to 85 and 60 to 80 years) for both conditions and compared against age-only and metadata-only AUC within each subgroup. For PD, AST-derived and metadata-based probabilities were additionally combined using equal-weight (0.5/0.5) soft voting with identical fold assignments.

#### Age-Restricted Retraining

To validate the post hoc age-restricted estimate, the AST was retrained from scratch using only participants aged 60 to 80 years (129 participants: 84 PD, 45 controls; PD: mean age 71.4, SD 5.8 years vs control: mean age 70.0, SD 6.5 years). The same architecture, hyperparameters, and 5-fold stratified cross-validation protocol were used. An age-only logistic regression was trained on the same cohort for comparison.

#### Country-Level Analysis

In the absence of explicit site identifiers in v3.0.0, participant country of origin (United States or Canada, from *demographics.tsv*) was used as a coarse proxy for institutional variation. The AST’s existing OOF predictions were stratified by country, and AUC-ROC was computed for each country subset. Age-restricted evaluation (ages 60 to 80 years) was performed within the US subset.

#### Attention Map Analysis

Classify token (CLS-token) attention weights were extracted from all 12 transformer layers of the fold-1 AST model for prolonged-vowel recordings (105 PD and 147 controls). Statistical testing included permutation tests, FDR-corrected pixel-wise comparisons, Cohen *d* effect sizes, and frequency-band analysis. Full methodology is described in Section 7 in Multimedia Appendix 1.

### Sensitivity and Supporting Analyses

A sensitivity analysis using all available speech tasks (rather than the 8 selected tasks) was conducted with the same AST architecture and evaluation protocol, with minor hyperparameter adjustments for the larger dataset (max epochs 20, patience 5, and constant learning rate). A foundation model comparison evaluated PPGs [25] processed through the same AST architecture, and a 1D CNN on native 40-dimensional PPGs, using identical cross-validation splits.

This study is reported in accordance with the TRIPOD+AI (Transparent Reporting of a Multivariable Prediction Model for Individual Prognosis or Diagnosis–Artificial Intelligence) guidelines for prediction model studies [16]. The completed checklist is provided in Checklist 1.

### Ethical Considerations

The Bridge2AI Voice Dataset v3.0.0 (primary) and v2.0.1 (preprocessing robustness testing) used in this study are publicly available through PhysioNet. Access requires credentialing through PhysioNet and execution of the Bridge2AI Voice Registered Access Data Use Agreement. Data collection and sharing were approved by the University of South Florida Institutional Review Board. As this study exclusively used deidentified, publicly available datasets accessed under a data use agreement, no additional institutional review board approval was required. Code for this analysis is available on GitHub [26].

## Results

### Cohort Characteristics and Demographic Imbalance

**Figure 3.**
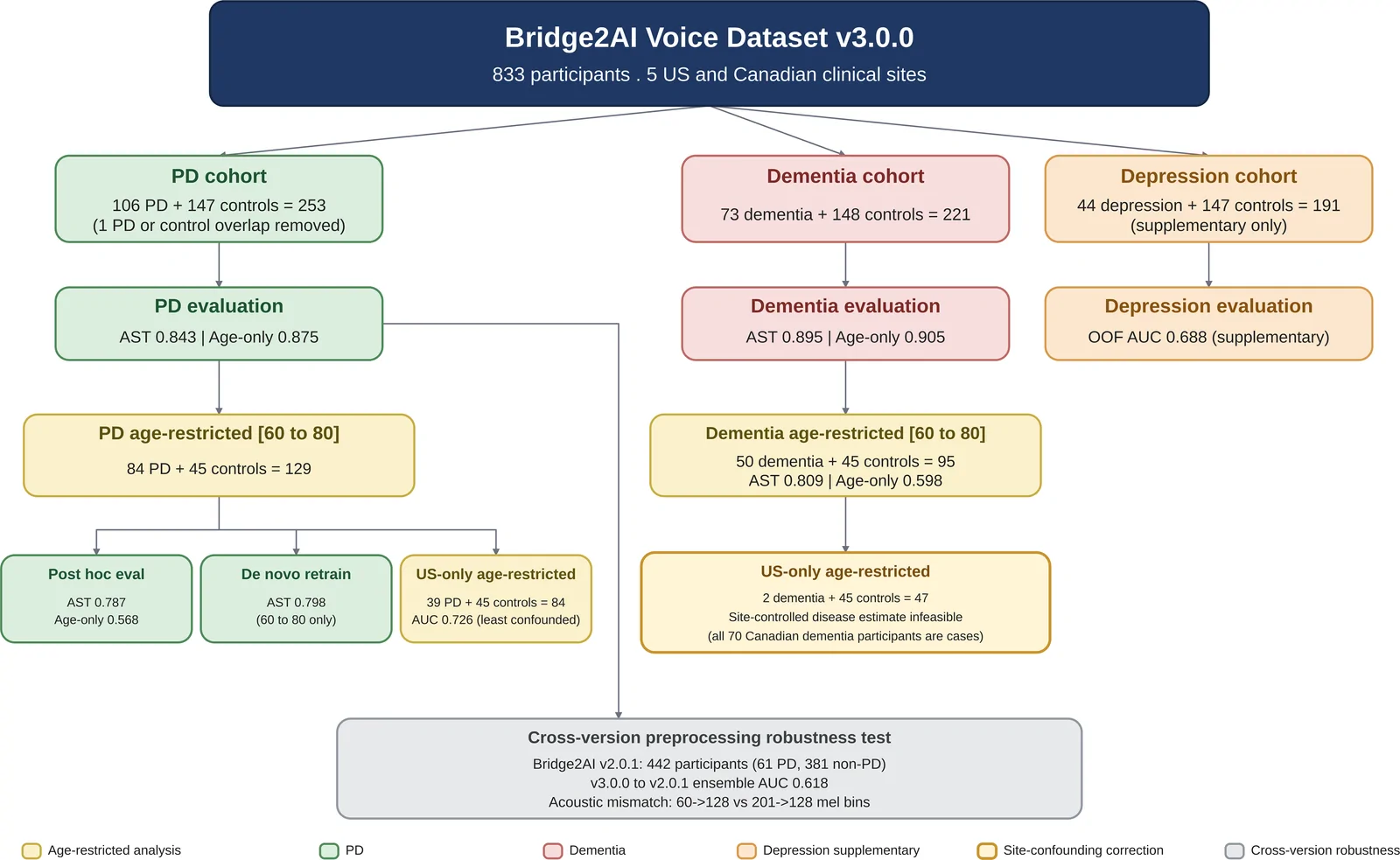
Participant flow diagram for condition-specific cohort construction from Bridge2AI Voice Dataset v3.0.0 (833 participants; 5 US and Canadian clinical sites). AST: audio spectrogram transformer; AUC: area under the curve; Bridge2AI: Bridge to Artificial Intelligence; OOF: out-of-fold; PD: Parkinson disease.

Condition-specific cohorts were constructed from the hierarchical phenotype annotations in Bridge2AI v3.0.0 (Figure 3). PD cases (106 participants) were identified from *parkinsons_disease.tsv* and dementia cases (73 participants) from *cognitive_impairment.tsv*. Controls (147 participants after removing 1 participant with both PD and control labels) were drawn from *control.tsv*. Comorbidity across conditions was minimal (1 participant with both PD and dementia; Table S4 in Multimedia Appendix 1), supporting the interpretation of condition-specific analyses. Eight speech tasks spanning sustained phonation, pitch control, articulatory agility, connected speech, and cognitive-linguistic domains were selected. Participant-level characteristics are summarized in Table 2. All results reported below are at the participant level, with predicted probabilities aggregated across recordings per individual. Depression screening (44 cases) is reported in Multimedia Appendix 1 due to the small sample size.

**Table 2.** Cohort composition and demographics for PD^a^ and dementia screening (Bridge2AI^b^ v3.0.0; 5 US and Canadian clinical sites)^c^.

Fields	Parkinson disease	Dementia
Participants	253	221
Cases and controls	106, 147	73^d^, 148
Recordings	2131	1791
Selected tasks	8	8
Case proportion (%)	41.9	33
Demographics		
Case age (years), mean (SD)	72.3 (8.7)	74.9 (7.6)
Control age (years), mean (SD)	44.8 (19.3)	44.7 (19.3)
Age gap (years)	27.5	30.2
Case male, n (%)	60 (56.6)	45 (61.6)
Control male, n (%)	70 (47.6)	71 (48.0)

a PD: Parkinson disease.

b Bridge2AI: Bridge to Artificial Intelligence.

c Both cohorts share the same control group (n=147‐148, mean age 44.7, SD 19.3 to mean 44.8, SD 19.3 years), producing case-control age gaps of 27.5 years (PD) and 30.2 years (dementia).

d One participant was excluded due to a comorbid Parkinson disease diagnosis.

Both cohorts exhibit striking demographic imbalances (Table 2). PD cases are on average 27.5 years older than controls (mean 72.3, SD 8.7 years vs mean 44.8, SD 19.3 years), and the dementia gap is even larger at 30.2 (mean 74.9, SD 7.6 vs mean 44.7, SD 19.3) years. This shared pattern arises because both conditions predominantly affect older adults, while the control group, common to both cohorts, skews young (mean 44.7, SD 19.3 years to mean 44.8, SD 19.3 years). Sex distributions are more balanced (56.6%‐61.6% vs 47.6%‐48%, male). This demographic structure is not unique to Bridge2AI; similar or unreported age imbalances characterize many voice-based disease screening datasets. The consequences of this imbalance for model performance are the central focus of this study.

### Age Confounding Dominates Screening Performance

To quantify the impact of the age imbalance on screening performance, we compared the fine-tuned AST against demographic-only classifiers using the same 5-fold cross-validation splits. The analysis was performed for both PD (252 participants with complete demographic data) and dementia (220 participants).

### PD

#### Age Alone Outperforms the AST

A logistic regression classifier using only participant age achieved an OOF AUC of 0.875 for PD classification, exceeding the fine-tuned AST (AUC 0.843), although DeLong test indicates the difference is not statistically significant (ΔAUC=–0.028, 95% CI –0.088 to +0.032; *P*=.36; q=0.462). Adding sex at birth provided no additional benefit (age+sex AUC 0.869; sex-only AUC 0.543; logistic regression coefficient approximately 0.1 for sex vs approximately 2.1 for age). This result demonstrates that the age gap between cases and controls is the primary source of discriminability in the full PD cohort: a model with no access to speech recordings outperforms an 86.4-million-parameter deep learning model trained on spectrograms.

#### Age-Restricted Subgroup Analysis Isolates Residual Disease Signal

To disentangle age-related voice changes from disease-specific features, we evaluated the AST’s existing OOF predictions on age-restricted subgroups where the age gap was minimized. Restricting to participants aged 55 to 85 years (151 participants: 102 PD, 49 controls; PD: mean age 73.0, SD 7.1 years vs control: mean age 68.9, SD 7.2 years) reduced age-only AUC from 0.875 to 0.655, while the AST maintained an AUC of 0.805. In the narrower 60 to 80 window (129 participants: 84 PD, 45 controls; PD: mean age 71.4, SD 5.8 years vs control: mean age 70.0, SD 6.5 years), age-only AUC dropped to 0.568 (near chance), while the AST retained an AUC of 0.787 (Table 3).

**Table 3.** Demographic confounding analysis for PD^a^ screening (Bridge2AI^b^ v3.0.0; 252 participants with complete demographics; 5-fold stratified cross-validation, participant-level, out-of-fold)^c,d,e,f,g^.

Model	Cohort	N	AUC-ROC^h^
Full cohort (age gap=27.5 years*)*			
Age only (raw)	All	252	0.875
Metadata only (age+sex)	All	252	0.869
AST^i^ (spectrogram)	All	252	0.843
AST+Metadata (soft voting)	All	252	0.924
Age-restricted subgroup (age gap=4.1 years)			
AST (spectrogram)	Ages 55‐85	151	0.805
Age only (raw)	Ages 55‐85	151	0.655
Metadata only (age+sex)	Ages 55‐85	151	0.641
Strictly age-restricted subgroup (age gap=1.4 years*)*			
AST (spectrogram)	Ages 60‐80	129	0.787
Age only (raw)	Ages 60‐80	129	0.568
Metadata only (age+sex)	Ages 60‐80	129	0.552
Age-restricted retraining (trained exclusively on 60‐80 subgroup)			
AST retrained (spectrogram)	Ages 60‐80	129	0.798
Age only (retrained cohort)	Ages 60‐80	129	0.555

a PD: Parkinson disease.

b Bridge2AI: Bridge to Artificial Intelligence.

c Bootstrap 95% CIs (2000 iterations): AST full cohort (0.791-0.890).

d AST age-restricted 60‐80 (0.695-0.863).

e AST retrained (0.710, 0.878).

f Age-only age-restricted (0.458, 0.667).

g Age-only AUC (0.875) exceeds the AST (0.843) on the full cohort; under age restriction (60‐80 years, n=129), age-only drops to 0.568 while the AST retains 0.787. Age-restricted retraining (bottom) yields 0.798.

h AUC-ROC: area under the receiver operating characteristic curve.

i AST: audio spectrogram transformer.

This age-restricted AUC of 0.787 (bootstrap 95% CI 0.695-0.863) represents the most credible estimate of the AST’s disease-specific discriminative capacity, as it is obtained under conditions where demographic confounding can no longer explain the result.

The pattern across progressively narrower age windows is consistent and interpretable: as the age gap narrows, the age-only classifier approaches chance performance, while the AST retains substantial discrimination. This demonstrates that the AST learns genuine disease-relevant acoustic features, but that these features account for an AUC of approximately 0.787, not the 0.843 reported on the full cohort. The AUC difference of 0.056 is attributable to age confounding. Late fusion of AST and metadata probabilities through equal-weight soft voting yielded a combined AUC of 0.924 on the full cohort, confirming that the AST captures complementary information beyond demographics.

#### Age-Restricted Retraining Suggests Disease-Specific Signal

To rule out the possibility that the post hoc subgroup estimate (0.787) reflects features learned from the age-confounded full cohort, we retrained the AST from scratch using only the 129 participants aged 60 to 80 years (84 PD and 45 controls; mean age gap 1.4 years). We acknowledge that fine-tuning all 86.4 million AST parameters on 129 participants carries substantial overfitting risk despite regularization measures (differential learning rate, focal loss, early stopping, and SpecAugment). Transfer learning from AudioSet pretraining mitigates this partially, but the effective number of freely optimized parameters relative to the sample size remains unfavorable. This retrained model achieved an OOF AUC of 0.798 (bootstrap 95% CI 0.710-0.878; mean fold AUC 0.805, SD 0.085), while age-only logistic regression on the same cohort yielded an AUC of 0.555 (near chance). Fold-level AUCs ranged from 0.667 to 0.889 (SD 0.085), indicating substantial instability. This high variance suggests the retrained model’s OOF AUC of 0.798 should be interpreted cautiously. The retrained AUC (0.798) closely matches the post hoc estimate (0.787), suggesting that the full-cohort model’s age-restricted performance reflects disease-specific features rather than residual age-correlated signal from training. This result directly addresses the concern that post hoc subgroup analysis may overestimate performance due to features learned during confounded training (Table 3).

#### Country-Level Analysis Reveals Site-Diagnosis Confounding

##### Overview

Bridge2AI v3.0.0 does not expose explicit site identifiers, but the country of origin (United States and Canada) provides a coarse proxy for institutional variation. Among the 253 PD cohort participants with demographic data, 191 are from the United States (44 PD and 147 controls) and 62 from Canada (62 PD and 0 controls). The complete absence of Canadian controls means that country perfectly predicts diagnosis for Canadian participants, introducing site-level confounding that compounds the age effect. Restricting to US-only participants, the AST achieved an OOF AUC of 0.779 (age gap 25.0 years); under age restriction (ages 60‐80 years, n=84: 39 PD, 45 controls; age gap 0.95 years), the US-only AUC was 0.726. The lower US-only age-restricted AUC (0.726) compared to the full-cohort age-restricted AUC (0.787) likely reflects the removal of the perfectly confounded Canadian subsample. These findings demonstrate that site-level demographic imbalance, here, an entire country contributing only cases, can further inflate screening performance beyond the age confound alone. All 62 Canadian participants in the PD cohort are PD cases with zero controls; similarly, all 70 Canadian dementia participants are cases. Consequently, models may partially learn country-of-origin rather than disease-specific features.

##### Propensity-Matched Analysis (PD)

Within the US-only ages 60‐80 years PD subgroup (n=84; 39 PD and 45 controls), 1:1 nearest-neighbor propensity-score matching on age and sex (logistic-regression propensity score; caliper 0.2×SD; no replacement) produced 28 matched pairs (72% of cases matched within caliper, 28/39); the postmatch standardized mean differences were 0.095 for age and 0.071 for sex, both below the conventional 0.1 balance threshold. For the matched cohort, predictions were the existing OOF probabilities from the full-cohort AST; no model was retrained on the matched subset. Matching therefore balances the evaluation distribution on age and sex but does not remove demographic structure that the model may have learned during full-cohort training. On this matched cohort (n=56), the AST achieved an AUC of 0.760 (95% CI 0.628-0.877), while age-only and metadata classifiers were at chance (0.504 and 0.499). DeLong test for AST versus age-only on the matched cohort yielded Δ AUC +0.256 (95% CI +0.038 to +0.475; *P*=.02; q=0.039). This converges with the post hoc age-restricted estimate (0.787) and the de novo retrained estimate (0.798). We note that the matched (0.760) and post hoc (0.787) estimates both reuse the full-cohort model’s OOF predictions and are therefore not mutually independent; the de novo retrained estimate (0.798), which never observed the confounded full cohort during training, is the independent corroboration. Taken together, the matched result shows that the AST’s discrimination persists on a cohort balanced on age and sex.

##### Dementia Propensity Matching Infeasible

The US-only ages 60‐80 years dementia subgroup contained only 2 cases (vs 45 controls), precluding propensity matching. The full age-restricted dementia AUC of 0.809 therefore necessarily includes Canadian participants, where 100% are cases. This means that our estimate is at least partially confounded by site-level case-control imbalance. We caveat the dementia disease-specific interpretation accordingly.

### Dementia

The dementia cohort exhibits an even larger age gap (30.2 years; cases: mean 74.9, SD 7.6 vs controls: mean 44.7, SD 19.3). Age-only logistic regression achieved an AUC of 0.905 for dementia classification, exceeding the AST’s 0.895, mirroring the PD pattern, although DeLong test indicates the difference is not statistically significant (ΔAUC=–0.009, 95% CI –0.067 to +0.049; *P*=.75; q=0.826; Table 4). Under age restriction (60‐80 years; 95 participants: 50 dementia, 45 controls; mean age gap 2.5 years), the AST retained an AUC of 0.809 while age-only classification dropped to 0.598. We note that all 70 Canadian dementia participants are cases with zero controls, identical in confounding structure to the PD cohort. The dementia age-restricted AUC of 0.809 is therefore particularly susceptible to site confounding and should be interpreted with corresponding caution.

**Table 4.** Demographic confounding analysis for dementia screening (Bridge2AI^a^ v3.0.0; 220 participants; 5-fold stratified cross-validation, participant-level, out-of-fold)^b^.

Model	Cohort	N	AUC-ROC^c^
Full cohort (age gap=30.2 years*)*			
Age only (raw)	All	220	0.905
Metadata only (age+sex)	All	220	0.901
AST^d^ (spectrogram)	All	220	0.895
Age-restricted subgroup (age gap=6.3 years*)*			
AST (spectrogram)	Ages 55‐85	121	0.839
Age only (raw)	Ages 55‐85	121	0.727
Strictly age-restricted subgroup (age gap=2.5 years*)*			
AST (spectrogram)	Ages 60‐80	95	0.809
Age only (raw)	Ages 60‐80	95	0.598
Metadata only (age+sex)	Ages 60‐80	95	0.587

a Bridge2AI: Bridge to Artificial Intelligence.

b Age-only AUC (0.905) exceeds the AST (0.895); under age restriction (60‐80 years, n=95), age-only drops to 0.598 while the AST retains 0.809.

c AUC-ROC: area under the receiver operating characteristic curve.

d AST: audio spectrogram transformer.

The consistency of these findings across 2 conditions sharing the same control group, both showing age-only AUC exceeding the AST and both showing complete Canadian site confounding (Canada contributes only cases), strongly suggests that demographic confounding is a systematic property of this dataset’s design rather than a condition-specific artifact. We emphasize that, unlike PD, the dementia age-restricted AUC (0.809) cannot be attributed to a disease-specific signal once site confounding is considered, because the US-only dementia subgroup contains too few cases to isolate it.

### Cross-Version Preprocessing Robustness

Bridge2AI v3.0.0 is a cumulative release that subsumes v2.0.1: session-level identifier matching confirms that 441 of 442 v2.0.1 participants reappear in v3.0.0 under reanonymized participant identifiers, consistent with the PhysioNet release notes (v3.0.0 adds “an additional 391 participants” to the 442 in v2.0.1). The two releases therefore represent the same participants recorded with the same voice tasks, but with spectrograms extracted through different pipelines (60 vs 201 Mel bins) and different phenotype annotation structures. To assess the sensitivity of learned representations to preprocessing, the 5-fold models trained on the 253-participant v3.0.0 spectrograms were applied as an ensemble to the v2.0.1 spectrograms of the same participants (442 participants: 61 PD and 381 non-PD).

v2.0.1 spectrograms were resized to (128 ×1024) and normalized using each fold’s training statistics. The ensemble achieved a participant-level AUC of 0.618, a substantial drop from both the full-cohort OOF AUC (0.843) and the age-restricted AUC (0.787). This performance drop likely reflects the acoustic mismatch between upsampled (v3.0.0, 60 to 128 bins) and downsampled (v2.0.1, 201 to 128 bins) spectrograms; the model was trained on characteristically smooth interpolation patterns absent from the v2.0.1 data, rather than a pure generalization failure.

Because v2.0.1 contains the same participants as a subset of v3.0.0, this drop cannot be attributed to population differences. Instead, it isolates the effect of spectrogram extraction: the model’s learned features do not transfer across preprocessing pipelines, suggesting sensitivity to low-level spectral representation rather than robust encoding of vocal biomarkers. This brittleness reinforces the broader concern that reported within-dataset performance may reflect artifacts of the specific feature extraction pipeline as much as genuine disease signal.

### AST Screening Performance

#### Overview

Having established the confounding context, we report the AST screening results (Table 5). Per-fold metrics are in Table S7 in Multimedia Appendix 1 and Figure S4 in Multimedia Appendix 1.

**Table 5.** Aggregated out-of-fold AST^a^ screening performance for PD^b^ and dementia (Bridge2AI^c^ v3.0.0; 5-fold stratified cross-validation, participant-level)^d^.

Metric	Parkinson disease	Dementia
Participants (cases and controls)	253 (106, 147)	221 (73, 148)
AUC-ROC^e^	0.843	0.895
Age-restricted AUC^f^ (60-80)	0.787	0.809
*F*_1_-score	0.686	0.773
Recall	0.679	0.863
Precision	0.692	0.700
LOFO^g^ threshold, mean (SD)	0.509 (0.034)	0.459 (0.030)

a AST: audio spectrogram transformer.

b PD: Parkinson disease.

c Bridge2AI: Bridge to Artificial Intelligence.

d Full-cohort areas under the curve are inflated by age confounding (27.5‐30.2 year gaps); age-restricted areas under the curve (Table 4) provide less confounded estimates.

e AUC-ROC: area under the receiver operating characteristic curve.

f AUC: area under the curve.

g LOFO: leave-one-fold-out.

#### PD

**Figure 4.**
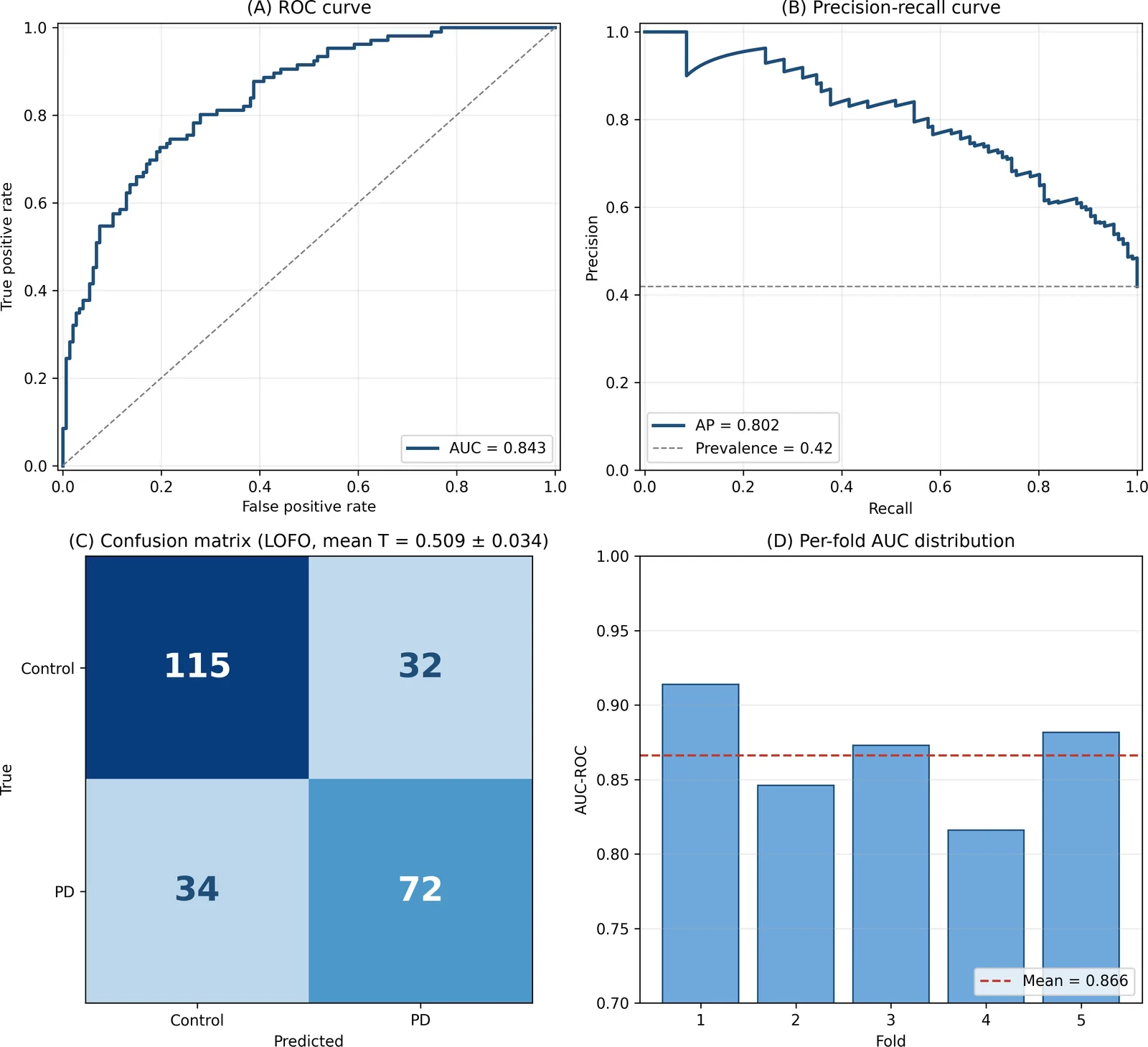
Participant-level audio spectrogram transformer classification performance for Parkinson disease (Bridge2AI v3.0.0; 253 participants; 5-fold stratified cross-validation). (A) Receiver operating characteristic curve (out-of-fold area under the curve 0.843; age-restricted estimate 0.787; Table 3). (B) Precision-recall curve. (C) Confusion matrix at the mean leave-one-fold-out threshold (0.509), selected by the Youden J statistic. (D) Per-fold area under the curve distribution (mean 0.866, SD 0.037). AP: average precision; AUC: area under the curve; AUC-ROC: area under the receiver operating characteristic curve; LOFO: leave-one-fold-out; PD: Parkinson disease; ROC: receiver operating characteristic.

Mean per-fold AUC-ROC was 0.866 (SD 0.037; 95% CI 0.820-0.912). Aggregated OOF predictions yielded an AUC-ROC of 0.843; under the LOFO threshold protocol (mean threshold 0.509, SD 0.034), *F*_1_-score=0.686, precision=0.692, recall=0.679, specificity=0.782 (Table S7 in Multimedia Appendix 1; Figure 4). Under the LOFO protocol, each fold's threshold was derived from the OOF predictions (Youden J statistic) of the remaining folds rather than from within-fold training partitions, so no participant's threshold depends on their own prediction. Because threshold selection still uses held-out predictions from other participants, the reported *F*_1_-score, precision, and recall remain slightly optimistic; AUC is threshold-independent and unaffected. As shown in the “Age Confounding Dominates Screening Performance” section, the age-restricted estimate (AUC 0.787) should be considered the more reliable indicator of disease-specific performance.

#### Dementia

Mean per-fold AUC-ROC was 0.901 (SD 0.032; 95% CI 0.862-0.940). Aggregated OOF evaluation produced an AUC-ROC of 0.895; under the LOFO threshold protocol (mean threshold 0.459, SD 0.030), *F*_1_-score=0.773, precision=0.700, recall=0.863, specificity=0.818. The full-cohort AUC is inflated by a 30.2-year age gap; the age-restricted estimate (AUC 0.809) is more reliable (see the “Age Confounding Dominates Screening Performance” section).

### Baseline Comparisons and Ablation

To contextualize the AST’s performance, additional baselines were evaluated on the PD cohort using identical cross-validation splits (Table S2 in Multimedia Appendix 1 and Figure S1 in Multimedia Appendix 1). A logistic regression baseline on spectrogram summary statistics achieved an OOF AUC of 0.585, a frozen AST baseline (linear probing; 0.23% of parameters trainable) achieved 0.776, and a ResNet18 convolutional baseline achieved 0.821. Fine-tuning improved over frozen representations by 0.067 AUC. The fine-tuned AST outperformed all speech-based baselines but was outperformed by age alone (0.875), underscoring the dominance of the demographic confounder.

An ablation removing SpecAugment during training yielded an OOF AUC of 0.846, nearly identical to 0.843 with augmentation (Δ=0.003). This near-identical performance with and without SpecAugment may indicate that the model relies on coarse spectral features potentially including those correlated with age or recording site, rather than fine-grained spectrotemporal patterns. SpecAugment masks localized time-frequency regions; if classification is driven by global spectral statistics (eg, overall energy distribution or fundamental frequency range), such masking would have minimal impact regardless of sample size.

### Supporting Analyses

A PPG [25] comparison showed that spectrogram-based representations outperformed phoneme-based inputs for both PD (0.843 vs 0.737) and dementia (0.895 vs 0.816); however, the PPG-AST comparison is confounded by the interpolation of 40-dimensional categorical probability vectors to 128 bins, which distorts the distributional semantics of the input. The lower PPG-AST performance may reflect this preprocessing artifact rather than an inherent architectural limitation. The 1D CNN on native 40-dimensional PPGs (AUC 0.786) avoids this issue and provides a fairer phoneme-level baseline (Table S9 in Multimedia Appendix 1). A sensitivity analysis using all available speech tasks (9331 recordings vs 2131 for selected tasks) yielded an OOF AUC of 0.942, though this elevated performance likely reflects task completion patterns acting as a data leak; the pattern of completed versus missing tasks may indirectly encode participant age and condition (Table S10 and Figure S5 in Multimedia Appendix 1). Both models showed reasonable calibration (PD Brier score 0.166; dementia 0.130; Table S5 in Multimedia Appendix 1; Figure S2 in Multimedia Appendix 1), and all 8 selected tasks exceeded chance-level PD discrimination (Figure S3 in Multimedia Appendix 1). Table 6 summarizes AST screening AUCs for PD across all analytical conditions reported above, from the full cohort (0.843) through age restriction and propensity matching (0.760) to the cross-version preprocessing and all-tasks sensitivity analyses.

**Table 6.** Summary of AST^a^ screening AUC^b^ across analytical conditions for Parkinson disease (Bridge2AI^c^ Voice Dataset v3.0.0; 5-fold stratified cross-validation, participant-level, out-of-fold)^d^.

Condition	N	AST AUC (95% CI)	Age-only AUC	DeLong P (BH^k^ q)
Full cohort	252	0.843 (0.791‐0.890)	0.875	.36 (0.462)^f^
Age-restricted (55-85)	151	0.805 (—^g^)	0.655	.007 (0.016)
Age-restricted (60-80)	129	0.787 (0.695‐0.863)	0.568	.001 (0.005)
Age-restricted retrained	129	0.798 (0.710‐0.878)	0.555	.002^h^
US-only full	191	0.779 (—)	—	—
US-only age-restricted	84	0.726 (—)	—	—
*Propensity-matched (United States, age + sex)*	*56*	*0.760 (0.628‐0.877)*	*0.504*	*.021 (0.039)*
Cross-version preprocessing (v2.0.1)	442	0.618 (—)	—	—
All available speech tasks	253	0.942 (—)	—	—

a AST: audio spectrogram transformer.

b AUC: area under the curve.

c Bridge2AI: Bridge to Artificial Intelligence.

d The italicized row marks the analysis with the strongest formal evidence of disease-specific signal beyond demographics (1:1 propensity matching on age and sex within the US-only ages 60‐80 Parkinson disease subgroup; AST vs age-only DeLong *P*=.02; q=0.039). AUC 95% CIs are bootstrap intervals (2000 resamples) where computed; q=Benjamini-Hochberg FDR-adjusted P. The ages 55‐85 row is a wider, overlapping version of the prespecified ages 60‐80 age restriction. The progression from 0.843 (full cohort) to 0.760 (matched) tracks the removal of demographic confounders. Cross-version preprocessing AUC (0.618) reflects spectrogram-pipeline sensitivity rather than population generalization; all-tasks AUC (0.942) suffers from task-completion missingness (Multimedia Appendix 1).

e BH: Benjamini-Hochberg.

f Not significant.

g Not applicable.

h De novo retrained audio spectrogram transformer versus age-only on the retrained cohort; reported for completeness and not part of the 9-comparison Benjamini-Hochberg family, hence shown uncorrected.

Attention map analysis of the fold-1 model (Section 7 in Multimedia Appendix 1 and Figure S6 in Multimedia Appendix 1) revealed no statistically significant global differences in attention patterns between PD and control groups (permutation test: *P*=.16 for the full cohort; *P*=.88 for the age-restricted subgroup). Exploratory frequency-band analysis identified elevated PD attention in the lowest frequency band (~0‐600 Hz; Cohen *d*=0.57), but this finding did not survive correction for the 6 bands tested and should be interpreted as hypothesis-generating rather than confirmatory.

## Discussion

### Principal Results

The central finding of this study is that age confounding dominates screening performance for both PD and dementia in the Bridge2AI Voice Dataset: a simple age threshold outperforms a fine-tuned 86.4-million-parameter AST for both conditions (age-only AUC 0.875 vs AST 0.843 for PD; 0.905 vs 0.895 for dementia). The consistency of this pattern across 2 conditions sharing the same control group strongly suggests that the confounding is a systematic property of the dataset’s demographic structure rather than a condition-specific artifact. This result challenges the interpretation of high classification metrics commonly reported in the voice-based disease screening literature and has broad implications for how studies in this field should be designed, evaluated, and reported.

### The Scope of Demographic Confounding

The finding that age alone achieves an AUC of 0.875 for PD classification, exceeding the AST’s 0.843, is striking but not surprising in retrospect. PD onset typically occurs at approximately 60 years of age [13], while volunteer control cohorts in voice research typically skew younger. The resulting age gap creates a statistical shortcut: any classifier that captures age-correlated features (including age-related voice changes such as decreased pitch, increased jitter, and reduced harmonic-to-noise ratio [14,15]) will achieve high discrimination without learning anything about PD itself. The dementia cohort confirms this is not condition-specific: with a 30.2-year age gap, age alone achieves 0.905, again exceeding the AST (0.895). Under age restriction (60 to 80 years), the dementia AST AUC was 0.809 while age-only dropped to 0.598; however, unlike PD, this age-restricted estimate cannot be interpreted as disease-specific, because all 70 Canadian dementia participants are cases and only 2 of 47 US-only ages 60‐80 years dementia participants are cases. The 0.809 figure therefore conflates disease signal with site and effectively functions as a country classifier, and no site-controlled dementia estimate is derivable from this dataset. Statistically, age-only and AST classifiers were not distinguishable in the full cohort for either condition (PD: *P*=.36, q=0.462; dementia: *P*=.75, q=0.826), meaning the AST contributes no measurable discrimination beyond demographic features at full-cohort level. Under age-restricted evaluation (60‐80 years), the AST exceeded age-only by a wide and statistically significant margin (PD: Δ AUC +0.225, 95% CI +0.089 to +0.361, *P*=.001, q=0.005; dementia: +0.215, 95% CI +0.056 to +0.375, *P*=.008, q=0.018), confirming that the model captures genuine disease-specific information that emerges only after the age confound is removed. We note that the PD and dementia cohorts share the same 148-person control group (mean age 44.7, SD 19.3 years). Consequently, the parallel confounding pattern across both conditions is a single structural observation, not 2 independent validations. The converging evidence is informative; it shows age predicts both conditions’ labels, but the shared controls mean both age-only baselines are mechanistically coupled.

This problem is not unique to the Bridge2AI dataset. Brenner et al [18] demonstrated that age-based selection bias inflates PD voice classification on the mPower dataset, and Hireš et al [27] showed that cross-dataset voice-based PD detection yields substantially degraded performance even when within-dataset accuracy is high, consistent with our cross-version finding (AUC 0.618). We have found “shortcuts” in imaging [28], and others have found demographic shortcuts that AI models often use [29]. Many PD voice studies use datasets with similar or unreported demographic structures. Rahman et al [21] achieved AUC 0.753 on a web-based speech task (726 participants; PD: mean age 65.9 years vs control: mean age 58.0 years) and acknowledged age and sex as confounders, but their mitigation was limited to excluding participants younger than 50 years; they did not test whether age alone could predict PD status. Of 13 representative studies surveyed (Table S12 in Multimedia Appendix 1 [9,10,20,30-36]), only 7 report age distributions for both cases and controls, none performed a deliberate age-restricted subgroup evaluation, and none tested a demographic-only baseline. Studies reporting 90% to 97% classification accuracy [8,34] typically do not assess the contribution of age to their results (Table S3 in Multimedia Appendix 1 [34,36,37]). Without such controls, it is impossible to determine what fraction of reported performance reflects genuine disease-specific signal versus demographic confounding. We note that the TRIPOD+AI guidelines [16] explicitly recommend assessment of demographic factors in prediction models, yet this analysis is largely absent from the voice biomarker literature.

From a clinical perspective, these findings urge caution in interpreting reported screening performance for voice-based PD and dementia tools. An AUC of 0.787 under age-restricted conditions is substantially lower than the 0.843 reported on the full cohort; yet, it reflects genuine disease-specific discriminative ability rather than demographic artifact.

The age-restricted subgroup analysis provides a practical solution. By restricting evaluation to participants aged 60 to 80 (PD: mean age 71.4, SD 5.8 vs control: mean age 70.0, SD 6.5) years, we effectively eliminate age as a confounder (age-only AUC drops to 0.568, near chance). Under these conditions, the AST retains an AUC of 0.787, suggesting that the model does capture genuine disease-specific acoustic features, but at a substantially lower level than the full-cohort metric suggests. Retraining the AST from scratch on the age-restricted subgroup corroborates this estimate (AUC 0.798), ruling out the concern that the post hoc result reflects features learned during confounded training. This convergence of post hoc evaluation (0.787) and de novo retraining (0.798) suggests that voice-based features may contain disease-relevant information at approximately this performance level, though confirmation requires prospective validation on independent, age-balanced cohorts.

The confounding problem extends beyond age to site-level demographic imbalance. All 62 Canadian participants in the PD cohort are cases (zero controls), creating perfect site-diagnosis confounding. Restricting to US-only participants under age restriction yields an AUC of 0.726, lower than the full-cohort age-restricted AUC of 0.787, suggesting that the perfectly confounded Canadian subsample inflates even the age-restricted metric. The complete case-control site confounding (Canada contributes only cases for both conditions) means the age-restricted AUC of 0.787 may still be partially inflated by site-specific acoustic properties. The US-only age-restricted estimate of 0.726, while lower, is arguably the least confounded performance estimate available from this dataset. We recommend this figure be given greater interpretive weight. This finding highlights that multisite datasets may introduce additional confounders that are not resolved by age restriction alone.

Cross-version preprocessing robustness testing reinforces the fragility of within-dataset metrics: performance dropped from 0.843 to 0.618 when v3.0.0-trained models were applied to v2.0.1 spectrograms of the same participants (442 individuals with spectrograms reextracted at 201 vs 60 Mel bins). Because v2.0.1 participants are a subset of v3.0.0 (confirmed via session identifier matching), this drop isolates the effect of the spectrogram extraction pipeline rather than population differences, revealing that the model’s learned representations are brittle to preprocessing changes, a concern for clinical deployment where feature extraction standardization cannot be guaranteed.

### Implications for the Field

The demographic confounding problem identified here likely extends beyond PD. Any condition that predominantly affects older or younger adults (eg, Alzheimer disease, amyotrophic lateral sclerosis, and attention-deficit disorders) will produce age-imbalanced datasets when cases are compared against convenience-sampled controls. Voice changes with age are well documented [15], creating a persistent confounder that standard evaluation metrics cannot resolve. Concurrent work achieving 0.89 AUC-ROC on Bridge2AI [36] has not been evaluated under age restriction, and self-supervised models [9,10] face the same concern.

We propose that future voice biomarker studies adopt three minimum reporting requirements: (1) report the age distribution of cases and controls, (2) train and evaluate a demographic-only baseline using the same cross-validation splits, and (3) report age-restricted subgroup performance alongside full-cohort metrics. These additions require minimal computation but would substantially improve interpretability. We further recommend that dataset creators prioritize age-restricted cohort designs at the recruitment stage.

The educational origin of these findings is instructive: the age confounding was first identified through bias auditing exercises designed for early-career researchers at the Emory Health AI Summer School & Datathon [22], suggesting that structured training in demographic bias assessment, rather than specialized methodological expertise, is sufficient to uncover these issues. This supports the integration of bias auditing protocols into standard machine learning training curricula for biomedical researchers.

### Limitations

The principal evidence for a residual disease-specific signal in this study comes from 3 subgroup analyses with substantially reduced sample sizes, and several sources of confounding could not be fully eliminated within the constraints of the available data. We organize the limitations below according to the analytical layer they affect.

### Sample Size and Statistical Interpretation

The principal evidence for a disease-specific PD signal beyond demographics rests on 3 convergent subgroup analyses: post hoc age-restricted evaluation (129 participants, AUC 0.787), de novo retraining on the age-restricted subgroup (the same 129, AUC 0.798), and 1:1 propensity-score matching on age and sex within the US-only ages 60 to 80 PD subgroup (28 matched pairs, AUC 0.760). The triangulation across these 3 procedures is the strongest argument that the AST captures information beyond demographics; we note, however, that only the de novo retraining is fully independent of the confounded full-cohort model, whereas the post hoc and propensity-matched estimates share the full-cohort OOF predictions, but each individual estimate has wide CIs (eg, bootstrap 95% CI 0.695-0.863 for the post hoc estimate) and the retrained model exhibited substantial fold-to-fold variance (SD 0.085) reflecting the unfavorable ratio of 86.4 million parameters to 129 participants. The corresponding statistical tests must also be interpreted in this context: the full-cohort DeLong tests were not statistically significant (PD: *P*=.36, q=0.462; dementia: *P*=.75, q=0.826), which is a failure to reject equivalence rather than a positive demonstration of it; the age-restricted superiority of the AST over age alone (PD: *P*=.001, q=0.005; dementia: *P=*.008, q=0.018) and the propensity-matched DeLong *P*=.02, q=0.039 are statistically significant but obtained on small samples and remain vulnerable to sample-specific effects. Replication on larger, prospectively recruited age-balanced cohorts is necessary, and future work should examine whether progressive layer freezing or adapter-based fine-tuning yields more stable estimates in the small-sample regime. A further source of optimism is that model checkpoints were selected by early stopping on each fold’s held-out partition, which was then aggregated to report OOF performance; this couples model selection to the evaluation set and biases the reported AUCs upward. We did not implement nested cross-validation owing to the computational cost of repeatedly fine-tuning an 86.4-million-parameter model. Critically, this bias inflates the AST’s metrics, not the demographic-only baselines (which use independent logistic-regression cross-validation); it therefore cannot explain why age alone matches or exceeds the AST on the full cohort, and the true disease-specific signal is, if anything, slightly lower than the age-restricted estimates reported here.

### Residual Site Confounding and Dementia Estimate

Although the US-only ages 60 to 80 years estimate (AUC 0.726) substantially reduces the site confound introduced by the all-case Canadian subsample, it does not eliminate it. Bridge2AI v3.0.0 does not expose explicit site identifiers, so we used country of origin as a coarse proxy; institutional, device, or recording-environment variation among the 4 US sites remains unmeasured and could still partially inflate the residual signal. We therefore present 0.726 as the most conservative estimate available from this dataset, not as an unconfounded one. The corresponding dementia analysis is more constrained still: only 2 of the 47 US-only ages 60 to 80 dementia participants were cases, which precluded propensity matching and means the dementia age-restricted AUC of 0.809 necessarily includes the entirely-case Canadian subsample. A properly site-adjusted dementia disease-specific estimate analogous to the PD estimate of 0.726 cannot be derived from this dataset, and demonstrating an unconfounded dementia voice signal will require a cohort with substantially more US-based dementia cases in the 60 to 80 age range.

### Demographic and Clinical Covariates Not Incorporated

Bridge2AI records participant race, ethnicity, primary language, education level, Hoehn-Yahr stage (PD), Montreal Cognitive Assessment scores, and medication state, none of which were incorporated into the present analysis. Voice biomarkers are known to encode race, accent, and language background, and the AST backbone was pretrained on AudioSet, which may carry its own demographic biases independent of any task-specific signal. Diagnoses were treated as binary, which means residual disease-specific performance may disproportionately reflect more severely affected cases and will not generalize uniformly across the disease severity spectrum. Medication effects, particularly levodopa for PD, were not controlled, and the dataset comprises only English-language speakers, limiting cross-linguistic generalizability. Severity-stratified, demographic-stratified, and medication status–stratified analyses are essential prerequisites for any clinical translation claim.

### Generalizability Beyond This Dataset and Architecture

The parallel confounding pattern observed across PD and dementia (which share the same control group) suggests a structural property of the Bridge2AI dataset rather than a condition-specific artifact, but the demographic-shortcut phenomenon was not quantified for other voice datasets in this study. Our survey of 13 published voice-PD studies (Table S12 in Multimedia Appendix 1) found that fewer than half report case and control age distributions and none performed a demographic-only baseline, so the broader scope of confounding in the field remains unmeasured. The confounding analysis also used a single fine-tuned AST backbone; whether self-supervised speech foundation models, multitask learners, or alternative spectrotemporal architectures exhibit the same age-shortcut sensitivity is an open question. The PPG-AST comparison reported here is itself confounded by the interpolation of categorical phoneme posteriors and so cannot adjudicate architectural suitability. A further generalizability concern is task aggregation: participant-level predictions were computed as unweighted means across the 8 selected tasks, with recording counts ranging from 247 to 290 per task, so participants who completed more tasks contributed to a different mixture of predictions than those who completed fewer. If task completion correlates with age, motor function, or cognitive status, some of the residual disease-specific signal could reflect missingness patterns rather than acoustic content, as the elevated all-tasks AUC (0.942, Table S10 in Multimedia Appendix 1) most parsimoniously suggests. Task-weighted aggregation and an age-restricted evaluation of the all-tasks pipeline are important next steps. The minimum-duration filter (recordings with fewer than 100 timeframes excluded) operated at the recording level and removed only 8 of 2139 selected-task recordings (0.4%); no participant was excluded entirely (253 of 253 retained). The excluded recordings were modestly skewed toward PD cases (5 of 8) with higher mean age (69.4 vs 53.7 years for the 3 excluded control recordings), consistent in direction with duration-related survivorship but negligible in magnitude. This exclusion is therefore a minor component of the broader task-missingness mechanism discussed above rather than an independent source of bias (Table S11 in Multimedia Appendix 1).

### Cross-Version

We further note that the v2.0.1 spectrograms were standardized using each fold’s v3.0.0 training statistics. Because the two versions differ in base Mel resolution (60 bins upsampled to 128 vs 201 bins downsampled to 128), their raw intensity distributions differ, so applying v3.0.0 normalization parameters to v2.0.1 inputs may itself amplify the domain shift. The reported AUC of 0.618 should therefore be read as a combined index of representational brittleness and normalization mismatch rather than population non-generalization alone.

### Future Directions

Having demonstrated that age-restricted retraining confirms disease-specific signal (AUC 0.798 vs post hoc 0.787), the most impactful follow-up would be prospectively designed age-restricted cohort recruitment, which would address the confounder at the data collection stage rather than requiring post hoc correction or subgroup analysis on underpowered samples. Our country-level analysis revealed complete site-diagnosis confounding for Canadian participants; access to explicit site identifiers within Bridge2AI (which spans 5 clinical sites) would enable more granular site-level confounding analysis and site-aware model development. A systematic reevaluation of published results in the field, applying the demographic baseline and age-restricted protocol to existing datasets and models, would quantify the scope of the confounding problem across the voice biomarker literature.

### Conclusions

This methodological audit of the Bridge2AI Voice Dataset v3.0.0 yields 3 findings that map directly onto the study’s stated aims. First, full-cohort screening performance on this dataset is dominated by demographic confounding: age alone is statistically indistinguishable from a fine-tuned 86.4-million-parameter AST for both PD (DeLong: *P*=.36; q=0.462) and dementia (*P*=.75; q=0.826), and a single national subsample (Canadian participants) consists entirely of cases for both conditions. Because the PD and dementia cohorts share the same 148-person control group, the parallel confounding pattern reflects a single dataset-level structural artifact rather than 2 independent confirmations.

Second, after age restriction, site control, and propensity-score matching on age and sex, residual PD performance ranges from AUC 0.726 to 0.798 and exceeds age-only baselines at the propensity-matched level (*P*=.02; q=0.039), suggesting a residual disease-specific acoustic signal at substantially lower performance than the 0.843 full-cohort metric implies. An analogous unconfounded dementia estimate could not be derived because only 2 of 47 US-only ages 60 to 80 years dementia participants were cases. Cross-version preprocessing testing further showed that the learned representations are brittle to changes in the spectrogram extraction pipeline (AUC 0.618), indicating that within-dataset performance partially reflects features of the specific feature-extraction pipeline rather than robust acoustic biomarkers.

Third, drawing on these findings and on a survey of 13 published voice-PD studies in which 7 reported case and control age distributions and none performed a demographic-only baseline, we propose 3 minimum reporting standards for voice biomarker research: case and control demographic distributions, demographic-only baselines under identical cross-validation splits, and age-restricted subgroup performance reported alongside full-cohort metrics. Independent prospective validation on age-balanced cohorts, with explicit site identifiers and harmonized acoustic protocols, will be required before voice-based PD or dementia screening can be considered for clinical translation.

## Supplementary material

10.2196/95609 Multimedia Appendix 1 Detailed preprocessing and model training procedures, baseline and ablation comparisons, calibration and threshold-sensitivity analyses, per-fold and per-task results, spectrogram versus phonetic posteriorgram foundation model comparison, a supplementary depression screening analysis, attention map analysis, and a survey of demographic reporting in voice-based Parkinson disease classification studies.

10.2196/95609 Checklist 1 TRIPOD+AI checklist.

## Abbreviations

1D: 1-dimensional
AST: audio spectrogram transformer
AUC: area under the curve
AUC-ROC: area under the receiver operating characteristic curve
Bridge2AI: Bridge to Artificial Intelligence
CIMDAR-HIVE: Developing a Hive Learning and Datathon Supported Course on Imaging and Multimodal Data for Resource-Limited Institutions
CNN: convolutional neural network
FDR: false discovery rate
GELU: Gaussian Error Linear Unit
LOFO: leave-one-fold-out
NIH: National Institutes of Health
OOF: out-of-fold
PD: Parkinson disease
PPG: phonetic posteriorgram
TRIPOD+AI: Transparent Reporting of a Multivariable Prediction Model for Individual Prognosis or Diagnosis–Artificial Intelligence
