# Supplementary material for: Demographic Confounding in Voice-Based Parkinson Disease Screening: Methodological Analysis of the Bridge2AI Voice Dataset
Source: J Med Internet Res. 2026 Aug 20;28:e95609. doi: 10.2196/95609 (PMC13492483; doi:10.2196/95609)
Supplement: Supplementary file 1 [file jmir-v28-e95609-s001.docx]

Supplementary Information

**Demographic Confounding in Voice-Based Parkinson**

**Disease Screening: Methodological Analysis of the Bridge2AI**

**Voice Dataset**

Shikhar Shukla, Parvati Naliyatthaliyazchayil, Judy Gichoya, Saptarshi Purkayastha

Table of Contents

S[1.	Detailed Methods	2](#_Toc228729199)

[1.1 Preprocessing Details	2](#_Toc228729200)

[1.2 Model Architecture and Training Hyperparameters	2](#_Toc228729201)

[1.3 PPG Preprocessing and 1D CNN Architecture	3](#_Toc228729202)

[1.4 Cross-Version Preprocessing Robustness Protocol	3](#_Toc228729203)

S[2.	Supplementary Figures	9](#_Toc228729206)

S[3.	Supplementary Tables	11](#_Toc228729207)

S[4.	Supplementary Depression Analysis	14](#_Toc228729208)

[4.1	v2.0.1 Depression Results	14](#_Toc228729209)

[4.2	v3.0.0 Depression Results	14](#_Toc228729210)

[4.3	Interpretation	14](#_Toc228729211)

S[5.	Additional Analyses	15](#_Toc228729212)

[5.1	Per-Fold Cross-Validation Details	15](#_Toc228729213)

[5.2	Foundation Model Comparison: Spectrogram vs PPG	16](#_Toc228729214)

[5.3	Sensitivity Analysis: Effect of Task Selection	17](#_Toc228729215)

S[6.	Attention Map Analysis	18](#_Toc228729216)

[6.1	Layer-Wise Divergence	18](#_Toc228729217)

[6.2	Global Statistical Testing	18](#_Toc228729218)

[6.3	Exploratory Frequency-Band Analysis	18](#_Toc228729219)

S[7.	Demographic Reporting in Voice-Based PD Studies	20](#_Toc228729220)

# S1. Detailed Methods

This section provides implementation details for the preprocessing, model architecture, training configuration, and cross-version preprocessing robustness protocol summarized in the main text.

## 1.1 Preprocessing Details

***Temporal standardization.*** All spectrograms passing the inclusion criterion (≥100 timeframes) were adjusted to a fixed length of 1,024 timeframes. For spectrograms shorter than 1,024 frames, reflect padding was applied along the temporal dimension. Reflect padding was selected over zero padding to preserve local spectro-temporal continuity at segment boundaries; zero padding would introduce artificial silence that creates condition-independent spectral energy discontinuities, potentially confounding model learning. For spectrograms longer than 1,024 frames, center cropping was performed, extracting a contiguous 1,024-frame segment symmetrically around the temporal midpoint.

***Frequency resizing.*** Following temporal standardization, spectrograms were resized from their native frequency resolution to 128 Mel bins along the frequency axis to match the expected input resolution of the pretrained AST model. For v3.0.0 spectrograms (60 Mel bins), this constituted up sampling; for v2.0.1 spectrograms (201 Mel bins, used in cross-version validation), this constituted down sampling. Resizing was performed using linear interpolation (scipy.ndimage.zoom, order = 1).

***Normalization.*** A single global z-score normalization was applied per fold: one scalar mean and one scalar standard deviation were computed over the entire fold-specific training partition (all training spectrograms pooled across all time-frequency bins) and then applied to every spectrogram in both the training and validation partitions of that fold. Statistics were therefore derived only from training data, so no information from validation samples influenced preprocessing. A small constant (1 × 10⁻⁸) was added to the denominator to ensure numerical stability.

***Final representation.*** After preprocessing, each spectrogram was represented as a normalized two-dimensional array of shape (128 Mel bins × 1,024 timeframes), treated as a grayscale image for input to the AST.

## 1.2 Model Architecture and Training Hyperparameters

***Architecture details.*** Input spectrograms of shape 128 × 1,024 were transposed to 1,024 × 128 to match AST input conventions, then internally divided into non-overlapping patches, each projected into a 768-dimensional embedding vector. Learned positional embeddings were added to preserve temporal and spectral context.

***Data augmentation.*** SpecAugment-inspired masking was applied randomly during training. Time masking selected a contiguous block of up to 150 frames and set the corresponding spectrogram values to zero, applied with 50% probability per sample. Frequency masking selected a contiguous block of up to 30 Mel bins and zeroed them, also applied with 50% probability. These augmentations were applied independently and only during training; validation spectrograms were left unmodified.

***Training configuration.*** Class weights were computed using the balanced formula w*c* = N*total* / (2 × n*c*), where N*total* is the total number of training recordings and n*c* is the count for class c. This recomputation was performed independently for each cross-validation fold to reflect the fold-specific class distribution. Model selection was based on a composite score combining participant-level AUC-ROC (weight 0.4) and optimized F1 score (weight 0.6), evaluated on the held-out partition after each epoch. Training was terminated early if no improvement exceeding 0.01 in the composite score was observed for 10 consecutive epochs.

**Table S1.** Training hyperparameters for the primary cross-validation analyses.

| **Hyperparameter** | **Value** |
| --- | --- |
| Optimizer | AdamW (β1 = 0.9, β2 = 0.999) |
| Backbone learning rate | 5 × 10⁻⁶ |
| Classification head learning rate | 5 × 10⁻⁴ |
| Weight decay | 0.01 |
| Batch size | 8 |
| Loss function | Focal loss (γ = 2.0) |
| Class weight computation | Inverse class frequency (per fold) |
| Learning rate schedule | Cosine annealing (ηmin = 1 × 10⁻⁷) |
| Gradient clipping | Max norm = 1.0 |
| Maximum epochs | 30 |
| Early stopping patience | 10 epochs |
| Early stopping criterion | 0.4 × AUC + 0.6 × F1opt |
| Model selection threshold | Δ > 0.01 in composite score |

## 1.3 PPG Preprocessing and 1D CNN Architecture

Posterior Phoneme Grams (PPGs) from features/ppgs.parquet (40 phoneme classes × variable time) were preprocessed identically to spectrograms: reflect-padded or center-cropped to 1,024 frames, resized to (128 × 1,024), and fold-normalized. The 1D CNN was evaluated under the identical participant-level 5-fold stratified cross-validation splits used for the AST (`StratifiedKFold`, shuffle=True, random_state=42), with predictions aggregated out-of-fold and AUC-ROC as the primary, threshold-independent metric. Secondary thresholded metrics used aggregated out-of-fold Youden's J; because the reported comparison metric is AUC, it is unaffected by threshold-selection strategy. Input PPGs were kept at their native 40-dimensional resolution (no interpolation to 128 bins), isolating the PPG-AST interpolation artifact from architectural effects.

A supplementary 1D CNN was evaluated on native 40-dimensional PPGs. The architecture comprised three convolutional layers (64, 128, and 256 filters respectively) with batch normalization and ReLU activation after each layer, followed by adaptive average pooling to a fixed-length representation and a linear classification head.

## 1.4 Cross-Version Preprocessing Robustness Protocol

To assess sensitivity of the trained models to changes in spectrogram extraction, a cross-version preprocessing robustness test was performed by applying v3.0.0-trained models to v2.0.1 spectrograms of the same participants. Because v2.0.1 participants are a subset of v3.0.0 (confirmed via session identifier matching), this test isolates the effect of the feature extraction pipeline (60 vs 201 Mel bins) from population-level differences. The five-fold-models from v3.0.0 cross-validation were applied as an ensemble (averaged predicted probabilities) to v2.0.1.

V2.0.1 spectrograms (201 Mel bins) were preprocessed with the same temporal standardization and resized to (128 × 1,024). Each fold’s normalization statistics (computed from v3.0.0 training data) were applied to the v2.0.1 spectrograms before prediction. Ensemble predictions were computed by averaging the five-fold-models’ predicted probabilities for each participant, and participant-level AUC-ROC was evaluated on the full v2.0.1 cohort (442 participants).

# S2. Supplementary Figures

**Figure S1. Baseline comparison of ROC curves for PD screening (selected tasks).** Participant-level out-of-fold receiver operating characteristic curves for models evaluated using identical five-fold cross-validation splits (v3.0.0).

**Figure S2. Calibration curves for PD and dementia.** Reliability diagrams and Brier scores computed from out-of-fold predictions of the fine-tuned AST on v3.0.0. Ten equal-width bins were used. The dashed diagonal represents perfect calibration. Lower Brier scores indicate better calibration (range 0–1).

**Figure S3. Per-task contribution to PD classification (v3.0.0).** Recording-level out-of-fold AUC for each of the eight selected speech tasks and the number of recordings per task (v3.0.0). All tasks exceeded chance-level discrimination (AUC *>* 0.5, dashed red line).

# S3. Supplementary Tables

**Table S2. Baseline and ablation comparison for PD screening (v3.0.0, selected tasks, participant-level, out-of-fold)**. All models used identical five-fold cross-validation splits (random state = 42) on the v3.0.0 PD cohort (253 participants: 106 PD, 147 controls).

| **Model** | **Trainable Parameters** | **OOF AUC** | **OOF  F1** | **OOF Recall** | **OOF  Precision** |
| --- | --- | --- | --- | --- | --- |
| Fine-tuned AST (no SpecAug) | 86.4M (100%) | **0.846** | **0.764** | 0.840 | 0.701 |
| Fine-tuned AST + SpecAug | 86.4M (100%) | 0.843 | 0.726 | 0.726 | 0.726 |
| ResNet18 (ImageNet) | 11.3M (100%) | 0.821 | 0.744 | 0.698 | **0.796** |
| Frozen AST (linear probe) | 199K (0.23%) | 0.776 | 0.710 | **0.868** | 0.601 |
| Logistic Regression | 810 | 0.585 | 0.456 | 0.368 | 0.600 |

^[1]^F1, recall, and precision for the baseline comparison are reported under the aggregated OOF Youden's J protocol used in the original development run; AUC-ROC is threshold-independent and unaffected. LOFO-corrected secondary metrics for the primary fine-tuned AST are reported in Table 5 of the main manuscript and Supplementary Table S8.

**Table S3. Cross-study comparison of voice-based screening approaches.** Direct numerical comparison is limited by differences in datasets, cohort sizes, evaluation protocols, and metrics. Studies using recording-level evaluation are expected to report higher performance due to within-subject correlation.

| **Study** | **Condition** | **Model** | **Dataset** | **N** | **Eval Level** | **Metric** |
| --- | --- | --- | --- | --- | --- | --- |
| This work | PD | AST (fine-tuned) | Bridge2AI v3.0.0 | 253 | Participant, OOF | AUC 0.843 (0.787 age-restricted) |
| This work | PD | PPG-AST | Bridge2AI v3.0.0 | 253 | Participant, OOF | AUC 0.737 |
| This work | PD | ResNet18 (CNN) | Bridge2AI  v3.0.0 | 253 | Participant, OOF | AUC 0.821 |
| This work | PD | AST (frozen) | Bridge2AI  v3.0.0 | 253 | Participant, OOF | AUC 0.776 |
| Amato 2021 | PD | AST | PC-GITA / ITA | 150 | Recording | 92-97% acc |
| MARVEL 2025 | Neurological | Multi-task | Bridge2AI v2.0 | 442 | Participant | AUROC 0.89 |
| This work | Dementia | AST (fine-tuned) | Bridge2AI v3.0.0 | 221 | Participant, OOF | AUC 0.895 |
| ADReSS 2020 | Dementia | Various | DementiaBank | ∼156 | Participant | ∼79% acc |

**Table S4. Comorbidity cross-tabulation across Bridge2AI v3.0.0 participants**. Labels derived from hierarchical phenotype annotations (N=360 participants with diagnosis data). Low overlap across conditions supports the interpretation that models learn condition-specific acoustic patterns rather than shared symptom profiles.

#### (a) Parkinson Disease × Dementia

|  | **Dementia+** | **Dementia**− | **Total** |
| --- | --- | --- | --- |
| PD+ | 1 | 105 | 106 |
| PD− | 73 | 181 | 254 |
| Total | 74 | 286 | 360 |

#### (b) Parkinson Disease × Depression

|  | **Depression+** | **Depression−** | **Total** |
| --- | --- | --- | --- |
| PD+ | 4 | 102 | 106 |
| PD− | 40 | 214 | 254 |
| Total | 44 | 316 | 360 |

#### (c) Dementia × Depression

|  | **Depression+** | **Depression−** | **Total** |
| --- | --- | --- | --- |
| Dementia+ | 5 | 69 | 74 |
| Dementia− | 39 | 247 | 286 |
| Total | 44 | 316 | 360 |

**Table S5. Calibration assessment (Brier scores) from out-of-fold predictions (v3.0.0)**. Lower scores indicate better calibration (range 0-1).

|  | **PD** | **Dementia** |
| --- | --- | --- |
| OOF AUC-ROC | 0.843 | 0.895 |
| Brier Score | 0.166 | 0.130 |
| Brier Score (Age-restricted 60–80) | 0.189 | -- |

**Table S6. Per-task recording distribution for PD classification (v3.0.0, selected tasks).** Recording counts for each task in the PD screening cohort (253 participants). All tasks were jointly modeled; per-task AUC decomposition is not available as participant-level predictions aggregate across tasks.

| **Task** | **Recordings** | **PD+** | **PD**− |
| --- | --- | --- | --- |
| Prolonged Vowel | 290 | 143 | 147 |
| Glides High-to-Low | 289 | 142 | 147 |
| Glides Low-to-High | 289 | 142 | 147 |
| Diadochokinesis (Pataka) | 254 | 129 | 125 |
| Rainbow Passage | 255 | 130 | 125 |
| Picture Description | 251 | 128 | 123 |
| Story Recall | 247 | 126 | 121 |
| Maximum Phonation Time | 256 | 131 | 125 |

# S4. Supplementary Depression Analysis

Depression screening was evaluated on both Bridge2AI v2.0.1 and v3.0.0 datasets. Due to the small sample size (44 cases in both versions), these results are reported as supplementary exploratory analyses rather than primary findings.

## 4.1 v2.0.1 Depression Results

Using 90 participants (44 depression, 46 controls) and 2 speech tasks (Animal Fluency, Open Response Questions), the fine-tuned AST achieved a mean cross-validated AUC-ROC of 0.666 (SD 0.236; 95% CI 0.373 to 0.959) with extreme fold-level variability (range 0.321-0.963). The aggregated OOF AUC-ROC was 0.632 with an optimized F1 of 0.699 (recall 0.818, precision 0.610). The substantial instability reflects the small cohort size (∼18 participants per validation fold) and limited recording volume (179 total from two tasks).

## 4.2 v3.0.0 Depression Results

Depression screening on v3.0.0 used 191 participants (44 depression, 147 controls) with tasks selected based on coverage of both depression and control participants (top 8 tasks by combined case-control representation). The same AST pipeline (5-fold StratifiedKFold, random state=42) was applied.

The fine-tuned AST achieved a mean cross-validated AUC-ROC of 0.754 (SD 0.076; 95% CI [0.659, 0.848]) with fold-level AUCs ranging from 0.663 to 0.847. The aggregated OOF AUC-ROC was 0.688 with an optimized F1 of 0.478 (recall 0.500, precision 0.458, threshold 0.668). Performance improved modestly compared to v2.0.1 (OOF AUC 0.688 vs 0.632), with reduced fold-level variance, likely attributable to the larger control group (147 vs 46) providing more stable cross-validation estimates.

## 4.3 Interpretation

The depression results across both dataset versions should be considered exploratory due to the persistent small sample size (44 cases). The extreme fold-level variance in v2.0.1 indicates that these results are not yet sufficiently stable to support clinical conclusions. The subtler acoustic manifestations of mood disorders, compared to the motor-speech impairments in PD or cognitive-linguistic changes in dementia, may require either more targeted speech elicitation tasks (e.g., emotional reading passages, mood-congruent narrative prompts) or complementary feature representations beyond log-Mel spectrograms.

# S5. Additional Analyses

## 5.1 Per-Fold Cross-Validation Details

Per-fold cross-validation metrics for PD and dementia are reported in Table S7. Per-fold AUC-ROC distributions are shown in Supplementary Figure S4.

**Table S7. Per-fold cross-validation summary (participant-level, optimal threshold, v3.0.0).** Mean, sample SD, and 95% CIs computed from n = 5 folds using the t-distribution.

|  | **Parkinson Disease** | | **Dementia** | |
| --- | --- | --- | --- | --- |
| Metric | Mean (SD) | 95% CI | Mean (SD) | 95% CI |
| AUC-ROC | 0.866 (0.037) | [0.820, 0.912] | 0.901 (0.032) | [0.862, 0.940] |
| F1 (optimal) | 0.811 (0.040) | [0.762, 0.860] | 0.843  (0.045) | [0.788, 0.899] |
| Recall | 0.905  (0.059) | [0.832, 0.978] | 0.918  (0.076) | [0.820, 1.008] |
| Precision | 0.741  (0.074) | [0.649, 0.832] | 0.785  (0.070) | [0.698, 0.872] |

**Figure S4. Per-fold AUC-ROC distribution by condition, showing fold-level stability (v3.0.0).**

5.1.1 **Threshold Strategies Comparison**

Table S8 contains the threshold-leakage sensitivity analysis for AST screening:

(1) Aggregated OOF Youden's J (the original protocol) selects the threshold that maximizes Youden's J on aggregated out-of-fold predictions, which leaks holdout labels into the threshold.

(2) Training-partition Youden's J selects the threshold per fold from the fold's own training-partition predictions, avoiding leakage but biased high for over-parametrized models that are overconfident on training data, visible especially for the dementia model (training threshold 0.71).

(3) LOFO Youden's J selects the threshold for fold k from out-of-fold predictions of folds {1..5} excluding k, providing leakage-free thresholds that remain calibrated on validation-distribution outputs.

The fixed = 0.5 row provides a no-optimization reference. The model is not threshold-sensitive: F1 varies by ≤0.04 across the three principled strategies for both conditions.

**Table S8. Threshold-leakage sensitivity analysis for Audio Spectrogram Transformer (AST) screening of Parkinson disease and dementia.** Secondary metrics (F1 score, precision, recall, and specificity) are reported under four threshold-selection strategies. LOFO Youden's J is the recommended leakage-free protocol; aggregated OOF Youden's J leaks holdout labels into the threshold and inflates secondary metrics. F1 varies by ≤0.04 across the three principled strategies, indicating the model is not threshold sensitive.

**PD (AUC = 0.843, N = 253)**

| **Strategy** | **Threshold** | **F1** | **Precision** | **Recall** | **Specificity** |
| --- | --- | --- | --- | --- | --- |
| Aggregated OOF Youden's J | 0.503 | 0.726 | 0.726 | 0.726 | 0.803 |
| Training-partition Youden's J (per fold) | 0.473 | 0.721 | 0.690 | 0.755 | — |
| LOFO Youden's J (recommended) | 0.509  (SD 0.034) | 0.686 | 0.692 | 0.679 | 0.782 |
| Fixed = 0.5 | 0.500 | 0.723 | 0.720 | 0.726 | 0.796 |

**Dementia (AUC = 0.895, N = 221)**

| **Strategy** | **Threshold** | **F1** | **Precision** | **Recall** | **Specificity** |
| --- | --- | --- | --- | --- | --- |
| Aggregated OOF Youden's J | 0.437 | 0.802 | 0.713 | 0.918 | 0.818 |
| Training-partition Youden's J (per fold) | 0.710 | 0.566 | 0.909 | 0.411 | 0.980 |
| LOFO Youden's J (recommended) | 0.459  (SD 0.030) | 0.773 | 0.700 | 0.863 | 0.818 |
| Fixed = 0.5 | 0.500 | 0.797 | 0.763 | 0.836 | 0.872 |

## 5.2 Foundation Model Comparison: Spectrogram vs PPG

Posterior Phoneme Grams (PPGs), encoding frame-level posterior probabilities over 40 phoneme classes, were processed through the same AST architecture with identical hyperparameters and cross-validation splits.

For PD, the PPG-AST achieved an OOF AUC of 0.737, compared to 0.843 for spectro-grams. For dementia, PPG-AST achieved 0.816 versus 0.895. A lightweight 1D CNN on native 40-dimensional PPGs achieved 0.786 for PD, outperforming the PPG-AST. The PPG-AST comparison is confounded by the interpolation of 40-dimensional categorical probability vectors to 128 bins, which distorts the distributional semantics of the input. The lower PPG-AST performance (PD: 0.737 vs 0.843; dementia: 0.816 vs 0.895) may reflect this preprocessing artifact rather than an inherent architectural limitation. The 1D CNN on native 40-dimensional PPGs (AUC 0.786) avoids this issue and provides a fairer phoneme-level baseline. The larger gap for PD is consistent with PD primarily affecting acoustic-motor speech production, which mel-spectrograms capture directly but phoneme-level abstractions partially discard. For dementia, where cognitive-linguistic features are more relevant, the PPG gap was smaller, suggesting phonetic representations retain more disease-relevant information.

**Table S9. Foundation model comparison: spectrogram-based AST vs PPG-based AST (v3.0.0, participant-level, out-of-fold).**

| **Model** | **PD OOF AUC** | **PD OOF  F1** | **Dementia OOF AUC** | **Dementia OOF F1** |
| --- | --- | --- | --- | --- |
| Spectrogram-AST | 0.843 | 0.726 | 0.895 | 0.802 |
| PPG-AST | 0.737 | 0.650 | 0.816 | 0.675 |
| PPG-1DCNN (PD only) | 0.786 | 0.695 | — | — |

## 5.3 Sensitivity Analysis: Effect of Task Selection

The same AST pipeline applied to all available speech tasks in v3.0.0 (9,331 recordings from 253 participants) yielded an OOF AUC of 0.942 (mean fold AUC 0.962, SD 0.033), compared to 0.843 for the eight selected tasks. The dramatic AUC increase from 0.843 (8 selected tasks) to 0.942 (all tasks) likely reflects task completion patterns acting as a data leak. In the all-tasks analysis, each participant contributes a variable number of recordings depending on which tasks they completed and whether recordings met the minimum duration threshold. If task completion is correlated with age, cognitive status, or motor function as is probable in a cohort spanning ages 18-90 with neurological conditions then the model can learn to classify based on which tasks are present rather than their acoustic content. This represents a form of indirect demographic encoding: the pattern of completed vs. missing tasks may encode participant age and condition more reliably than any individual spectrogram. We did not perform an age-restricted evaluation of the all-tasks model, which would help clarify whether the performance increase persists after controlling for demographic confounding. This omission is a limitation.

**Table S10. Comparison of PD screening performance: selected tasks versus all available tasks (v3.0.0).**

| **Metric** | **Selected Tasks** | **All Tasks** |
| --- | --- | --- |
| Participants (Case / Control) | 253 (106 / 147) | 253 (106 / 147) |
| Recordings | 2,131 | 9,331 |
| Mean AUC-ROC (SD) [95% CI] | 0.866 (0.037)  [0.820, 0.912] | 0.962 (0.033)  [0.921, 1.000] |
| OOF AUC-ROC | 0.843 | 0.942 |

**Figure S5.** PD screening: selected tasks vs. all tasks (v3.0.0).

## 5.4  Recording-Duration Exclusion and Survivorship Check

**Table S11. Recordings excluded by the 100-timeframe minimum (n_frames < 100), within the 8 selected PD-cohort tasks. Total selected-task recordings = 2,139 (2,131 retained, 8 excluded = 0.4%); no participant was removed entirely.**

| **Task** | **Control excluded** | **PD excluded** | **Total** |
| --- | --- | --- | --- |
| diadochokinesis-pataka | 0 | 1 | 1 |
| glides-high-to-low | 0 | 1 | 1 |
| glides-low-to-high | 0 | 1 | 1 |
| story-recall | 3 | 2 | 5 |
| **Total** | **3** | **5** | **8** |

# S6. Attention Map Analysis

To investigate which spectro-temporal regions drive PD classification, we extracted CLS-token attention weights from all 12 transformer layers of the fold-1 AST model for prolonged-vowel recordings (one per participant; 105 PD, 147 controls). Attention vectors were reshaped to a 6 × 202 patch grid (frequency × time) corresponding to the AST's internal patch decomposition of the 128 × 1024 input.

## 6.1 Layer-Wise Divergence

Mean absolute attention differences between PD and control groups increased progressively from layer 1 to layer 12 (Supplementary Figure S6), with the deepest layer showing over 300× greater divergence than the first. Deeper layers thus appear to encode increasingly disease-specific features.

## 6.2 Global Statistical Testing

A permutation test (N=1000) comparing overall PD vs control attention patterns did not reach significance (*P*=.163), and no individual grid positions survived FDR correction (0/1,212). Mean effect sizes were small (mean |d| = 0.094). Under age restriction (60-80; 84 PD, 45 controls), the global permutation P-value was .881, consistent with either age-driven attention differences or insufficient statistical power in the age-restricted subsample.

## 6.3 Exploratory Frequency-Band Analysis

Because the global permutation test was non-significant, band-specific results are presented as exploratory observations subject to multiple comparison inflation. Frequency-band analysis of the last layer identified elevated PD attention in the lowest band (~0-600 Hz, covering the fundamental frequency range): PD cases received 48% more attention than controls (t = 4.39, Cohen's d = 0.57). However, this finding did not survive correction for the six bands tested. Higher frequency bands showed no significant differences.

These results indicate that the AST does not show statistically significant global attention differences between PD and control groups, and the exploratory low-frequency band finding should be treated as hypothesis-generating rather than confirmatory. The non-significance under age restriction (*P*=.881) further suggests that attention patterns may reflect age-related rather than disease-specific features.

Attention was characterized on the fold-1 model. Because the global permutation test was non-significant (P=.163 for the full cohort and P=.881 for the age-restricted cohort) and no patch survived FDR or Bonferroni correction (0 of 1,212), the qualitative conclusion that there are no reliable PD versus control attention differences does not depend on the choice of fold. We therefore did not extract per-fold attention maps, and the low-frequency band difference is reported as exploratory and hypothesis-generating only.

**Figure S6. Enhanced AST attention analysis for PD classification (prolonged vowel, CLS token, fold 1).** Top row: mean attention maps (PD, Control, Difference). Middle row: Cohen’s d effect size map, layer-wise attention divergence across all 12 transformer layers (red = max divergence), and frequency-band analysis showing per-band mean attention difference (PD minus control); bands are shown in a single neutral color because no band survived correction for the six bands tested (lowest band covers ∼0-600 Hz / F0 range). Bottom row: age-restricted Cohen’s d, age-restricted difference, and permutation test null distributions (full cohort *P*=.163; age-restricted *P*=.881).

# S7. Demographic Reporting in Voice-Based PD Studies

Table S12 summarizes the demographic reporting practices across 13 representative voice-based PD classification studies, plus this work. For each study, we report the dataset used, sample size, age distributions (where available), whether age-restricted evaluation was performed, whether a demographic-only baseline was tested, and the evaluation granularity.

Of 13 studies surveyed, only 7 report age distributions for both cases and controls. Only 2 used age-restricted datasets by design (PC-GITA and the Jeong 2024 Korean corpus); none performed a deliberate age-restricted subgroup evaluation as a methodological control. No study prior to ours tested a demographic-only baseline to quantify the contribution of age to classification performance. Most studies evaluate at the recording level, meaning individual recordings from the same participant may appear in both training and test sets, further inflating performance estimates.

**Table S12. Demographic reporting in voice-based PD classification studies.** “NR” = not reported; “Inherited” = age matching inherited from the dataset design rather than performed as a deliberate evaluation step.

| **Study** | **Dataset** | **N (PD/Ctrl)** | **PD Age** | **Ctrl Age** | **Gap (y)** | **Age Restricted** | **Demo BL** | **Eval Level** |
| --- | --- | --- | --- | --- | --- | --- | --- | --- |
| Orozco-Arroyave 2014 | PC-GITA | 100 (50/50) | 62.2±11.2 | 61.2±11.3 | ~1 | Yesᵃ | No | Rec |
| Sakar 2013 | UCI PD Speech | 40 (20/20) | 65.4±8.8 | 48.2±21.6 | ~17 | No | No | Rec |
| Wroge 2018 | Custom | ~58 | NR | NR | NR | No | No | Rec |
| Tracy 2020 | mPower | Large | NRᵇ | NRᵇ | NR | No | Partial | Ptcp |
| Amato 2021 | PC-GITA + ITA | 150 (78/72) | ~62ᶜ | ~61ᶜ | ~1ᶜ | Inherited | No | Rec |
| Jeong 2024 | Korean custom | 200 (100/100) | 64.3±9.4 | 65.8±7.6 | ~1.5 | ~Yesᵃ | No | Rec |
| Laquatra 2024 | PC-GITA | 100 (50/50) | NR | NR | Matchedᶜ | Inherited | No | Rec |
| Chen 2025 | Hospital (China) | 131 (61/70) | 62±9 | 60±10 | ~2 | No | No | Rec |
| Quamar 2025 | Figshare | 81 (40/41) | NRᵈ | NRᵈ | NR | No | No | Rec |
| Sedigh 2025 | NeuroVoz | 112 (54/58) | 71.1±10.6 | 64.0±10.3 | ~7 | No | No | Rec |
| Piao 2025 | Bridge2AI v2 | 442 (61/381) | NR | NR | NR | No | No | Ptcp |
| Brenner 2023 | mPower | Large | NRᵇ | NRᵇ | NR | No | No | Rec |
| Rahman 2021 | Custom (web) | 726 (262/464) | 65.9±9.2 | 58.0±14.2 | ~8 | Partial | No | Ptcp |
| **This study** | **Bridge2AI v3** | **253 (106/147)** | **72.3±8.7** | **44.8±19.3** | **27.5** | **Yes** | **Yes** | **Ptcp** |

^a^ Age-restricted by dataset design, not as a deliberate evaluation step. ^b^ Demographics available in mPower metadata but not reported in the paper’s evaluation. ^c^ Inherited from PC-GITA; not independently reported for the AST evaluation. ^d^ Overall mean age 57.3 reported; per-group breakdown not provided.

Rec = recording-level; Ptcp = participant-level; Demo BL = demographic-only baseline tested; Age Match = deliberate age-restricted evaluation performed.
