# Supplementary material for: Demographic Confounding in Voice-Based Parkinson Disease Screening: Methodological Analysis of the Bridge2AI Voice Dataset
Source: J Med Internet Res. 2026 Aug 20;28:e95609. doi: 10.2196/95609 (PMC13492483; doi:10.2196/95609)
Supplement: Supplementary file 2 [file jmir-v28-e95609-s002.docx]

# TRIPOD+AI Checklist

This study is reported in accordance with the TRIPOD+AI guidelines for prediction model studies (Collins GS, et al. TRIPOD+AI statement: updated guidance for reporting clinical prediction models that use regression or machine learning methods. *BMJ*. 2024;385:e078378).

**Study type: Development with internal validation (5-fold cross-validation) and cross-version preprocessing robustness testing.**

| **Item** | **Section** | **Type** | **Checklist Item** | **Reported (Page/Section)** | |
| --- | --- | --- | --- | --- | --- |
| 1 | Title | D;E | Identify the study as developing or evaluating the performance of a multivariable prediction model, the target population, and the outcome to be predicted | Title (p. 1). Title identifies AST-based disease detection model for neurological/psychiatric conditions. | |
| 2 | Abstract | D;E | See TRIPOD+AI for Abstracts checklist | Abstract (p. 2). See Section 2.1 | |
| 3a | Introduction: Background | D;E | Explain the healthcare context and rationale for developing the prediction model, including references to existing models | Introduction (pp. 4-5). Diagnostic/screening context; speech as  non-invasive biomarker; existing approaches referenced. | |
| 3b | Introduction: Target population | D;E | Describe the target population and the intended purpose of the prediction model | Introduction (pp. 4-5). Target: individuals with PD or dementia (depression in Supplementary); purpose: screening for further clinical evaluation. | |
| 3c | Introduction: Health inequalities | D;E | Describe any known health inequalities between sociodemographic groups | Introduction (pp. 4-5);  Discussion (pp. 17-19). Speech biomarkers may vary with age, sex, language, and cultural background; noted as area for future investigation. | |
| 4 | Introduction: Objectives | D;E | Specify the study objectives, including whether development or validation | Introduction (pp. 4-5). Development with internal validation (5-fold CV) for PD and dementia, plus cross-version preprocessing robustness testing and foundation model comparison. | |
| 5a | Methods: Data sources | D;E | Describe the sources of data, rationale, and representativeness | Methods (Methods,  pp. 6-7): Dataset Description. Bridge2AI Voice Dataset v3.0.0 (primary) and v2.0.1 (cross-version preprocessing robustness)  from PhysioNet. Multi-site, publicly available. | |
| 5b | Methods: Dates | D;E | Specify the dates of the collected participant data | Data collection dates described in the parent dataset publications [21, 22]. | |
| 6a | Methods: Study setting | D;E | Specify key elements of the study setting | Methods (pp.6-7). Multi-site dataset; specific site details in parent publications  [21, 22]. | |
| 6b | Methods: Eligibility | D;E | Describe the eligibility criteria for study participants | Methods: Task Selection (pp.6-7). Spectrograms ≥100 time frames from selected  high-prevalence tasks. | |
| 6c | Methods: Treatments | D;E | Give details of any treatments received | Medication status not controlled. Noted as limitation (Discussion, p.19). | |
| 7 | Methods: Data preparation | D;E | Describe any data pre-processing and quality checking | Methods: Preprocessing (pp. 7-8). Four-step pipeline: temporal standardization, frequency resizing, z-score normalization, final representation. Same for all participants. | |
| 8a | Methods: Outcome definition | D;E | Clearly define the outcome being predicted and the time horizon | Methods (pp. 6-7). Binary classification from phenotype labels.  Cross-sectional (no time horizon). | |
| 8b | Methods: Outcome assessors | D;E | Describe qualifications of outcome assessors if subjective | N/A. Labels are self-reported condition status from phenotype file [21, 22]  (Methods, p. 6). | |
| 8c | Methods: Blinding | D;E | Report any actions to blind assessment of the outcome | Methods: CV (p. 8). Strict participant-level separation; OOF predictions from models that never observed the participant. | |
| 9a | Methods: Predictor selection | D | Describe the choice of initial predictors | Methods (pp. 6-7); Results (pp. 10-16). Full log-Mel spectrograms; no  manual feature selection. LR baseline: 809 summary statistics. | |
| 9b | Methods: Predictor definition | D;E | Clearly define all predictors | Methods (pp. 6–7).  Spectrograms: 128×1024 Mel-frequency representations. | |
| 9c | Methods: Predictor assessors | D;E | Describe qualifications of predictor assessors if subjective | N/A. Spectrograms are algorithmically derived; no subjective interpretation. | |
| 10 | Methods: Sample size | D;E | Explain how the study size was arrived at | Methods (pp. 10-11); Discussion: Limitations (p. 21). Determined by available dataset. No formal sample size calculation. Modest sizes acknowledged as limitation. | |
| 11 | Methods: Missing data | D;E | Describe how missing data were handled | Methods (pp. 6-9).  Spectrograms *<*100 frames excluded. Complete cases only. NaN in extracted features replaced with 0. | |
| 12a | Methods: Data use | D | Describe how data were used and partitioned | Methods (p. 8). Preliminary 80/20 split for development only; all reported results from 5-fold CV on full cohort. | |
| 12b | Methods: Predictor handling | D | Describe how predictors were handled | Methods (p. 7).  Z-score normalization per fold (training statistics only). StandardScaler for LR baseline. | |
| 12c | Methods: Model type | D | Specify type of model, rationale, building steps, hyperparameter tuning, internal validation | Methods (p. 8). AST (ViT backbone, AudioSet pretrained). AdamW, differential LR, focal loss, SpecAugment, early stopping. 5-fold CV. Fixed hyperparameters. | |
| 12d | Methods: Heterogeneity | D;E | Describe how heterogeneity in model performance was handled across clusters | Results (p. 15-18). Per-fold metrics with mean, SD, 95% CI. Fold-level variability discussed. No multi-centre analysis. | |
| 12e | Methods: Performance measures | D;E | Specify all measures and plots used | Methods (pp. 8-9); Results (pp. 11-16).  AUC-ROC, F1, precision, recall, Brier scores, calibration curves, ROC/PR curves, confusion matrices. | |
| 12f | Methods: Model updating | E | Describe any model updating | N/A. Development study; no updating performed. | |
| 12g | Methods: Predictions | E | Describe how predictions were calculated | N/A (development). Full code publicly available on GitHub (Code Availability, p. 20). | |
| 13 | Methods:  Class  imbalance | D;E | State why and how class imbalance  was addressed | Methods: Training (p. 8). Weighted  random sampler, focal loss with dynamic class weights (*w_c_* = *N_total_/*(2 × *n_c_*)),  class weight=‘balanced’ for LR. No post-hoc recalibration. | |
| 14 | Methods:  Fairness | D;E | Describe approaches used to  address model fairness | Results (pp. 11-16); Discussion (pp. 17-20). Addressed through demographic confounding analysis for both PD (age-only AUC 0.875 vs AST 0.843; 27.5-year age gap) and dementia (age-only 0.905 vs AST 0.895; 30.2-year gap). Age-restricted subgroup analysis (ages 60-80) isolates disease-specific signal (PD: AST 0.787 vs age-only 0.568; dementia: AST 0.809 vs age-only 0.598).  Country-level site confounding also assessed. No formal fairness assessment across racial/ethnic groups. | |
| 15 | Methods:  Model output | D | Specify output of the prediction  model and threshold identification | Methods (pp. 13). Participant-level probability (mean of recording-level softmax). Thresholds via Youden’s J on OOF predictions. | |
| 16 | Methods: Dev  vs. eval | D;E | Identify differences between  development and evaluation data | Methods (pp. 7–8). Primary: 5-fold CV on  v3.0.0. Cross-version preprocessing robustness testing between v3.0.0 and v2.0.1. Fold-specific normalization. | |
| 17 | Methods:  Ethics | D;E | Name ethics committee that  approved the study | Data Availability (p. 10). USF IRB approved parent study. De-identified public  data; no additional IRB required. | |
| 18a | Open Science:  Funding | D;E | Give source of funding and role of  funders | Acknowledgments (p. 20). NIH, RWJF, RSNA, Lacuna Fund, Moore Foundation  grants listed. | |
| 18b | Open Science: Conflicts | D;E | Declare conflicts of interest | Competing Interests (p. 21). P.N. employed at ConcertAI (independent); others declare none. |  |
| 18c | Open Science: Protocol | D;E | Indicate where protocol can be accessed | No formal study protocol was prepared prior to the analysis (Study Protocol and Registration, p. 21). |  |
| 18d | Open Science: Registration | D;E | Provide registration information | This study was not pre-registered in a prediction model registry (Study Protocol and Registration, p. 21). |  |
| 18e | Open Science: Data | D;E | Provide details of data availability | Data Availability (p. 20). PhysioNet, DOI: 10.13026/3xt6-rf05. |  |
| 18f | Open Science: Code | D;E | Provide details of code availability | Code Availability (p. 20). GitHub: Amorfati123/bridge2ai-voice-parkinsons-ast. |  |
| 19 | Patient/Public Involvement | D;E | Provide details of PPI or state no involvement | No patient or public involvement in the design, conduct, reporting, or dissemination of this research (Patient and Public Involvement, p. 14). |  |
| 20a | Results: Participant flow | D;E | Describe flow of participants | Results: Cohort Characteristics (pp. 10-11); Figure 3 (p. 10). 833 total (v3.0.0); condition-specific cohorts described with exclusion criteria. |  |
| 20b | Results: Characteristics | D;E | Report characteristics overall and for each setting | Results: Table 3 (p. 11). Case/control distributions, recording counts. |  |
| 21 | Results: Sample sizes | D;E | Specify number of participants and events in each analysis | Results (pp. 11-14). Per-fold train/val splits reported. PD cohort: 253 participants.  Dementia cohort: 221 participants. All-tasks sensitivity: 253 participants. |  |
| 22 | Results: Model specification | D | Provide details of full prediction model | Code Availability (p. 20); Methods (pp. 8-9). Architecture, checkpoint, and code all publicly available. |  |
| 23a | Results: Performance | D;E | Report performance estimates with confidence intervals | Results: Tables 4-7 (pp. 12–16). Per-fold and OOF metrics with SD. Bootstrap 95% CIs (2,000 iterations) for key AUC estimates.  Baseline comparison table. |  |
| 23b | Results: Heterogeneity | D;E | Report heterogeneity in model performance | Results (p. 11); Supplementary Depression Analysis. Per-fold variability reported. Depression (Supplementary): fold-level variability (AUC 0.663-0.847). |  |
| 24 | Results: Updating | E | Report results from any model updating | N/A. Development study; no updating performed. |  |
| 25 | Discussion: Interpretation | D;E | Give overall interpretation including fairness | Discussion (pp. 17-19). Comprehensive interpretation covering confounding scope (PD and dementia), cross-version preprocessing robustness, field implications, prior work context, and honest assessment of attention analysis significance. |  |
| 26 | Discussion: Limitations | D;E | Discuss limitations and effects on biases, uncertainty, and generalizability | Discussion: Limitations (p. 19). Seven limitations discussed including  age-restricted sample size, post-hoc vs retrained estimates, cross-version preprocessing robustness, cohort size, task selection, language/medication, and dementia post-hoc limitation. |  |
| 27a | Discussion: Input quality | D | Describe handling of poor quality or unavailable input data | Methods (p. 7); Discussion (pp. 17, 18). Minimum 100-frame threshold. Discussion notes that behavior on degraded recordings is uncharacterized. |  |
| 27b | Discussion: User interaction | D | Specify user interaction requirements and expertise level | Discussion (pp. 17-19). Automated pipeline: clinical interpretation of outputs requires clinical expertise. |  |
| 27c | Discussion: Next steps | D;E | Discuss next steps for future research | Discussion (pp. 17-19). Interpretability, cross-lingual evaluation, longitudinal monitoring, multi-modal fusion. |  |

## 1.1 TRIPOD+AI for Abstracts Checklist

| **Item** | **Requirement** | **Reported?** | **Location** |
| --- | --- | --- | --- |
| Study design | Identify whether the study developed or validated a prediction model | Yes | “We applied a pretrained Audio Spectrogram Transformer” |
| Participants | Describe the study participants and setting | Yes | “Bridge2AI Voice Dataset” with condition-specific cohorts |
| Outcome | State the outcome to be predicted | Yes | PD, dementia, depression screening |
| Predictors | Describe the predictors | Yes | “spectrogram-based” AST framework |
| Sample size | Report number of participants and outcome events | Yes | Cohort sizes implied from AUC reports; not explicitly stated |
| Model type | Describe the type of prediction model | Yes | “Audio Spectrogram Transformer (AST)” |
| Performance | Provide performance measures with confidence intervals | Yes | OOF AUC reported for all conditions and baselines; bootstrap 95% CIs now reported in the abstract for the key PD age-restricted, de novo retrained, and propensity-matched estimates |
| Conclusions | Provide an overall interpretation | Yes | Recommend three minimum reporting requirements: demographic distributions, demographic-only baselines, and age-restricted subgroup evaluation, as standards for voice biomarker research. |
